# Convergence and quasi-optimality of adaptive FEM with inhomogeneous Dirichlet data^☆^

**DOI:** 10.1016/j.cam.2013.06.009

**Published:** 2014-01-01

**Authors:** M. Feischl, M. Page, D. Praetorius

**Affiliations:** Institute for Analysis and Scientific Computing, Vienna University of Technology, Wiedner Hauptstraße 8-10, A-1040 Wien, Austria

**Keywords:** Adaptive finite element methods, Convergence analysis, Quasi-optimality, Inhomogeneous Dirichlet data

## Abstract

We consider the solution of a second order elliptic PDE with inhomogeneous Dirichlet data by means of adaptive lowest-order FEM. As is usually done in practice, the given Dirichlet data are discretized by nodal interpolation. As model example serves the Poisson equation with mixed Dirichlet–Neumann boundary conditions. For error estimation, we use an edge-based residual error estimator which replaces the volume residual contributions by edge oscillations. For 2D, we prove convergence of the adaptive algorithm even with optimal convergence rate. For 2D and 3D, we show convergence if the nodal interpolation operator is replaced by the $L^{2}$-projection or the Scott–Zhang quasi-interpolation operator. As a byproduct of the proof, we show that the Scott–Zhang operator converges pointwise to a limiting operator as the mesh is locally refined. This property might be of independent interest besides the current application. Finally, numerical experiments conclude the work.

## 1 Introduction

### 1.1 Model problem

By now, the thorough mathematical understanding of convergence and quasi-optimality of h-adaptive FEM for second-order elliptic PDEs has matured. However, the focus of the numerical analysis usually lies on model problems with homogeneous Dirichlet conditions, i.e.  −Δu=f in Ω with u=0 on Γ=∂Ω; see e.g.  [1–5]. On a bounded Lipschitz domain $\Omega \subset R^{2}$ with polygonal boundary Γ=∂Ω, we consider

(1) $$\begin{array}{ccc} -\Delta u & = & f\;\text{in }\Omega \text{,}\\ u & = & g\;\text{on }\Gamma_{D}\text{,}\\ \partial_{n}u & = & \phi \;\text{on }\Gamma_{N} \end{array}$$

with mixed Dirichlet–Neumann boundary conditions. The boundary Γ is split into two relatively open boundary parts, namely the Dirichlet boundary $\Gamma_{D}$ and the Neumann boundary $\Gamma_{N}$, i.e.  $\Gamma_{D}\cap \Gamma_{N}=0̸$ and ${\bar{\Gamma }}_{D}\cup {\bar{\Gamma }}_{N}=\Gamma$. We assume the surface measure of the Dirichlet boundary to be positive $\left|\Gamma_{D}\right|>0$, whereas $\Gamma_{N}$ is allowed to be empty. The given data formally satisfy $f\in {\overset{˜}{H}}^{-1}\left(\Omega \right)$, $g\in H^{1/2}\left(\Gamma_{D}\right)$, and $\phi \in H^{-1/2}\left(\Gamma_{N}\right)$. As is usually required to derive (localized) a posteriori error estimators, we assume additional regularity of the given data, namely $f\in L^{2}\left(\Omega \right)$, $g\in H^{1}\left(\Gamma_{D}\right)$, and $\phi \in L^{2}\left(\Gamma_{N}\right)$.

Whereas certain work on a posteriori error estimation for  (1) has been done, cf.  [6,7], none of the proposed adaptive algorithms have been proven to converge. While the inclusion of inhomogeneous Neumann conditions ϕ into the convergence analysis seems to be obvious, incorporating inhomogeneous Dirichlet conditions g is technically more demanding and requires novel ideas. First, discrete finite element functions cannot satisfy general inhomogeneous Dirichlet conditions. Therefore, the adaptive algorithm has to deal with an additional discretization $g_{\ell }$ of g. Second, this additional error has to be controlled in the natural trace space which is the fractional-order Sobolev space $H^{1/2}\left(\Gamma_{D}\right)$. Since the $H^{1/2}$-norm is non-local, the a posteriori error analysis requires appropriate localization techniques. These have recently been developed in the context of adaptive boundary element methods  [8–13]: Under certain orthogonality properties of $g-g_{\ell }\in H^{1}\left(\Gamma_{D}\right)$, the natural trace norm ${\left\|g-g_{\ell }\right\|}_{H^{1/2}\left(\Gamma_{D}\right)}$ is bounded by a locally weighted $H^{1}$-seminorm ${\left\|h_{\ell }^{1/2}{\left(g-g_{\ell }\right)}^{\prime }\right\|}_{L^{2}\left(\Gamma_{D}\right)}$. Here, $h_{\ell }$ is the local mesh-width, and ${\left(\cdot \right)}^{\prime }$ denotes the arc length derivative. Finally, in contrast to homogeneous Dirichlet conditions g=0, we loose the Galerkin orthogonality in the energy norm. This leads to certain technicalities to derive a contractive quasi-error which is equivalent to the overall Galerkin error in $H^{1}\left(\Omega \right)$. In conclusion, quasi-optimality and even plain convergence of adaptive FEM with non-homogeneous Dirichlet data is a nontrivial task. To the best of our knowledge, only  [14] analyzes convergence of adaptive FEM with inhomogeneous Dirichlet data. While the authors also consider the 2D model problem  (1) with $\Gamma_{D}=\Gamma$ and lowest-order elements, their analysis relies on an artificial non-standard marking criterion. Quasi-optimal convergence rates are not analyzed and can hardly be expected in general  [1].

It is well-known that the Poisson problem  (1) admits a unique weak solution $u\in H^{1}\left(\Omega \right)$ with u=g on $\Gamma_{D}$ in the sense of traces which solves the variational formulation

(2) $${\langle \nabla u,\nabla v\rangle }_{\Omega }={\langle f,v\rangle }_{\Omega }+{\langle \phi ,v\rangle }_{\Gamma_{N}}\;\text{for all }v\in H_{D}^{1}\left(\Omega \right)\text{.}$$

Here, the test space reads $H_{D}^{1}\left(\Omega \right)=\left\{v\in H^{1}\left(\Omega \right):v=0\text{ on }\Gamma_{D}\text{ in the sense of traces}\right\}$, and 〈⋅,⋅〉 denotes the respective$L^{2}$-scalar products.

### 1.2 Discretization

For the Galerkin discretization, let $T_{\ell }$ be a regular triangulation of Ω into triangles $T\in T_{\ell }$. We use lowest-order conforming elements, where the ansatz space reads

(3) $$S^{1}\left(T_{\ell }\right)=\left\{V_{\ell }\in C\left(\bar{\Omega }\right):V_{\ell }{|}_{T}\text{ is affine for all }T\in T_{\ell }\right\}\text{.}$$

Since a discrete function $U_{\ell }\in S^{1}\left(T_{\ell }\right)$ cannot satisfy general continuous Dirichlet conditions, we have to discretize the given data $g\in H^{1}\left(\Gamma_{D}\right)$. According to the Sobolev inequality on the 1D manifold $\Gamma_{D}$, the given Dirichlet data are continuous on ${\bar{\Gamma }}_{D}$. Therefore, the nodal interpoland $g_{\ell }$ of g is well-defined. As is usually done in practice, we approximate $g\approx g_{\ell }$. Again, it is well-known that there is a unique $U_{\ell }\in S^{1}\left(T_{\ell }\right)$ with $U_{\ell }=g_{\ell }$ on $\Gamma_{D}$ which solves the Galerkin formulation

(4) $${\langle \nabla U_{\ell },\nabla V_{\ell }\rangle }_{\Omega }={\langle f,V_{\ell }\rangle }_{\Omega }+{\langle \phi ,V_{\ell }\rangle }_{\Gamma_{N}}\;\text{for all }V_{\ell }\in S_{D}^{1}\left(T_{\ell }\right)\text{.}$$

Here, the test space is given by $S_{D}^{1}\left(T_{\ell }\right)=S^{1}\left(T_{\ell }\right)\cap H_{D}^{1}\left(\Omega \right)=\left\{V_{\ell }\in S^{1}\left(T_{\ell }\right):V_{\ell }=0\text{ on }\Gamma_{D}\right\}$.

### 1.3 A posteriori error estimation

An element-based residual error estimator for this discretization reads

(5) $$\rho_{\ell }^{2}=\underset{T\in T_{\ell }}{\sum }\rho_{\ell }{\left(T\right)}^{2}$$

with corresponding refinement indicators

(6) $$\rho_{\ell }{\left(T\right)}^{2}≔\left|T\right|\;{\left\|f\right\|}_{L^{2}\left(T\right)}^{2}+{\left|T\right|}^{1/2}\left({\left\|\left[\partial_{n}U_{\ell }\right]\right\|}_{L^{2}\left(\partial T\cap \Omega \right)}^{2}+{\left\|\phi -\partial_{n}U_{\ell }\right\|}_{L^{2}\left(\partial T\cap \Gamma_{N}\right)}^{2}+{\left\|{\left(g-g_{\ell }\right)}^{\prime }\right\|}_{L^{2}\left(\partial T\cap \Gamma_{D}\right)}^{2}\right)\text{,}$$

where [⋅] denotes the jump across edges. We prove reliability and efficiency of $\rho_{\ell }$ (Proposition 2) and discrete local reliability (Proposition 3). Inspired by Carstensen and Verfürth  [15] as well as Page and Praetorius  [16], we introduce an edge-based error estimator $\varrho_{\ell }$ which reads

(7) $$\varrho_{\ell }^{2}=\underset{E\in E_{\ell }}{\sum }\varrho_{\ell }{\left(E\right)}^{2}\text{.}$$

For an edge $E\in E_{\ell }$, its local contributions read

(8) $$\varrho_{\ell }{\left(E\right)}^{2}=\left\{ \begin{array}{cc} \left|E\right|{\left\|\left[\partial_{n}U_{\ell }\right]\right\|}_{L^{2}\left(E\right)}^{2}+\left|\omega_{\ell ,E}\right|{\left\|f-f_{\omega_{\ell ,E}}\right\|}_{\omega_{\ell ,E}}^{2} & \text{if }E\subset \Omega \text{,}\\ \left|E\right|{\left\|\phi -\partial_{n}U_{\ell }\right\|}_{L^{2}\left(E\right)}^{2} & \text{if }E\subseteq \Gamma_{N}\text{,}\\ \left|E\right|{\left\|{\left(g-g_{\ell }\right)}^{\prime }\right\|}_{L^{2}\left(E\right)}^{2} & \text{if }E\subseteq \Gamma_{D}\text{.} \end{array}\right.$$

Here, $\omega_{\ell ,E}\subset \Omega$ denotes the edge patch, and $f_{\omega_{\ell ,E}}$ denotes the corresponding integral mean. The advantage of $\varrho_{\ell }$ is that the volume residual terms ${\left|T\right|}^{1/2}{\left\|f\right\|}_{L^{2}\left(T\right)}$ in  (6) are replaced by the edge oscillations ${\left|\omega_{\ell ,E}\right|}^{1/2}{\left\|f-f_{\omega_{\ell ,E}}\right\|}_{\omega_{\ell ,E}}$, which are generically of higher order. The choice of $\left|E\right|{\left\|{\left(g-g_{\ell }\right)}^{\prime }\right\|}_{L^{2}\left(E\right)}^{2}$ to measure the contribution of the Dirichlet data approximation is influenced by the Dirichlet data oscillations, cf. Section  3.1. We prove that $\rho_{\ell }$ and $\varrho_{\ell }$ are locally equivalent (Lemma 4) and thus obtain reliability and efficiency of $\varrho_{\ell }$ (Proposition 5) as well as discrete local reliability (Proposition 6).

### 1.4 Adaptive algorithm

We use the local contributions of $\varrho_{\ell }$ to mark edges for refinement in a realization (Algorithm 7) of the standard adaptive loop (AFEM) (9) Our adaptive algorithm uses the well-studied Dörfler marking  [2] to mark certain edges for refinement. We stress, however, that all results also hold for a variant of the standard marking strategy, proposed in  [17,18], and we refer to the extended preprint  [19] for precise statements and proofs. Throughout, we use newest vertex bisection, and at least marked edges are bisected. Given some initial mesh $T_{0}$, the algorithm generates successively locally refined meshes $T_{\ell }$ with corresponding discrete solutions $U_{\ell }\in S^{1}\left(T_{\ell }\right)$ of  (4).

### 1.5 Main results

The first main result (Theorem 11) states that the adaptive algorithm leads to a contraction

(10) $$\Delta_{\ell +1}\leq \kappa \;\Delta_{\ell }\;\text{for all }\ell \in N_{0}\;\text{and some constant}\;0<\kappa <1$$

for some quasi-error quantity $\Delta_{\ell }≃\varrho_{\ell }^{2}$ which is equivalent to the error estimator. In particular, this proves linear convergence of the adaptively generated solutions $U_{\ell }\in S^{1}\left(T_{\ell }\right)$ to the (unknown) weak solution $u\in H^{1}\left(\Omega \right)$ of  (2). The main ingredients of the proof are an equivalent error estimator ${\overset{˜}{\varrho }}_{\ell }≃\varrho_{\ell }$ for which we prove some estimator reduction

(11) $${\overset{˜}{\varrho }}_{\ell +1}^{\;2}\leq q\;{\overset{˜}{\varrho }}_{\ell }^{\;2}+C\;{\left\|\nabla \left(U_{\ell +1}-U_{\ell }\right)\right\|}_{L^{2}\left(\Omega \right)}^{2}\;\text{for all }\ell \in N_{0}\;\text{and some}\;0<\kappa <1\;\text{and}\;C>0\text{,}$$

see Lemma 9, and a quasi-Galerkin orthogonality in Lemma 10, whereas the general concept follows that of  [1].

The second main result is Theorem 14 which states that the outcome of the adaptive algorithm is quasi-optimal in the sense of Stevenson  [5]: Provided the given data $\left(f,g,\phi \right)\in L^{2}\left(\Omega \right)\times H^{1}\left(\Gamma_{D}\right)\times L^{2}\left(\Gamma_{N}\right)$ and the corresponding weak solution $u\in H^{1}\left(\Omega \right)$ of  (2) belong to the approximation class

(12) $$A_{s}≔\left\{\left(u,f,g,\phi \right):{\left\|\left(u,f,g,\phi \right)\right\|}_{A_{s}}≔\underset{N\in N}{\sup}\left(N^{s}\sigma \left(N,u,f,g,\phi \right)\right)<\infty \right\}$$

with

(13) $$\sigma {\left(N,u,f,g,\phi \right)}^{2}≔\underset{T_{*}\in T_{N}}{\inf}\left\{\underset{W_{*}\in S^{1}\left(T_{*}\right)}{\inf}\overset{2}{\underset{L^{2}\left(\Omega \right)}{\left\|\nabla \left(u-W_{*}\right)\right\|}}+\overset{2}{\underset{D,*}{\mathrm{osc}}}+\overset{2}{\underset{T,*}{\mathrm{osc}}}+\overset{2}{\underset{N,*}{\mathrm{osc}}}\right\}\text{,}$$

the adaptively generated solutions also yield convergence order $O\left(N^{-s}\right)$, i.e.

(14) $${\left\|u-U_{\ell }\right\|}_{H^{1}\left(\Omega \right)}≲{\left({\left\|\nabla \left(u-U_{\ell }\right)\right\|}_{L^{2}\left(\Omega \right)}^{2}+{\mathrm{osc}}_{D,\ell }^{2}\right)}^{1/2}≲{\left(\#T_{\ell }-\#T_{0}\right)}^{-s}\text{.}$$

Here, $T_{N}$ denotes the set of all triangulations $T_{*}$ which can be obtained by local refinement of the initial mesh $T_{0}$ such that $\#T_{*}-\#T_{0}\leq N$. Moreover, ${\mathrm{osc}}_{T,*},{\mathrm{osc}}_{D,*}$, and ${\mathrm{osc}}_{N,*}$ denote the data oscillations of the volume data f, the Dirichlet data g, and the Neumann data ϕ, see Section  3.1.

The ingredients for the proof are the observation that the proposed marking strategy is optimal (Proposition 12) and the Céa-type estimate

(15) $${\left\|\nabla \left(u-U_{\ell }\right)\right\|}_{L^{2}\left(\Omega \right)}^{2}+{\mathrm{osc}}_{D,\ell }^{2}\leq C_{\mathrm{cea}}\left(\underset{W_{\ell }\in S^{1}\left(T_{\ell }\right)}{\inf}\overset{2}{\underset{L^{2}\left(\Omega \right)}{\left\|\nabla \left(u-W_{\ell }\right)\right\|}}+{\mathrm{osc}}_{D,\ell }^{2}\right)$$

for the Galerkin solution $U_{\ell }\in S^{1}\left(T_{\ell }\right)$ in Lemma 13.

For 3D, nodal interpolation of the Dirichlet data $g\in H^{1}\left(\Gamma \right)$ is not well-defined. In the literature, it is proposed to discretize g by use of the $L^{2}$-projection  [6] or the Scott–Zhang projection  [7]. Our third theorem (Theorem 17) states convergence of the adaptive algorithm for either choice in 2D as well as 3D. The proof relies on the analytical observation that, under adaptive mesh-refinement, the Scott–Zhang projection converges pointwise to a limiting operator (Lemma 15), which might be of independent interest. Finally, we stress that the same results (Theorems 11, 14 and 17) hold if the element-based estimator $\rho_{\ell }$ from  (5) to (6) instead of the edge-based estimator $\varrho_{\ell }$ is used and if Algorithm 7 marks certain elements for refinement.

### 1.6 Outline

The remainder of this paper is organized as follows: We first collect some necessary preliminaries on, e.g., newest vertex bisection (Section  2.2) and the Scott–Zhang quasi-interpolation operator (Section  2.3). Section  3 contains the analysis of the a posteriori error estimators $\rho_{\ell }$ from  (5) to (6) and $\varrho_{\ell }$ from  (7) to (8). Moreover, we state the adaptive Algorithm in Section  3.4. The convergence is shown in Section  4, while the quasi-optimality results are found in Section  5. Whereas the major part of the paper is concerned with the 2D model problem, Section  6 considers convergence of AFEM for 3D. Finally, some numerical experiments conclude the work.

## 2 Preliminaries

### 2.1 Notation

Throughout, $T_{\ell }$ denotes a regular triangulation which is obtained by ℓ steps of (local) newest vertex bisection for a given initial triangulation $T_{0}$. By $K_{\ell }≔K_{\ell }^{\Omega }\cup K_{\ell }^{\Gamma }$, we denote the set of all interior nodes, respectively the set of all boundary nodes of $T_{\ell }$. By $E_{\ell }$, we denote the set of all edges of $T_{\ell }$ which is split into the interior edges $E_{\ell }^{\Omega }=\left\{E\in E_{\ell }:E\cap \Omega \neq 0̸\right\}$ and boundary edges $E_{\ell }^{\Gamma }=E_{\ell }\setminus E_{\ell }^{\Omega }$. We restrict ourselves to meshes $T_{\ell }$ such that each $T\in T_{\ell }$ has an interior node, i.e. $\partial T\cap K_{\ell }^{\Omega }\neq 0̸$. Note, that this is only an assumption on the initial mesh $T_{0}$. We assume that the partition of Γ into Dirichlet boundary $\Gamma_{D}$ and Neumann boundary $\Gamma_{N}$ is resolved, i.e.  $E_{\ell }^{\Gamma }$ is split into $E_{\ell }^{D}=\left\{E\in E_{\ell }:E\subseteq {\bar{\Gamma }}_{D}\right\}$ and $E_{\ell }^{N}=\left\{E\in E_{\ell }:E\subseteq {\bar{\Gamma }}_{N}\right\}$. Note that $E_{\ell }^{D}$ (resp.  $E_{\ell }^{N}$) provides a partition of $\Gamma_{D}$ (resp.  $\Gamma_{N}$).

For a node $z\in K_{\ell }$, the corresponding patch is defined by

(16) $$\omega_{\ell ,z}=⋃\left\{T\in T_{\ell }:z\in \partial T\right\}\text{.}$$

For an edge $E\in E_{\ell }$, the edge patch is defined by

(17) $$\omega_{\ell ,E}=⋃\left\{T\in T_{\ell }:E\subset \partial T\right\}\text{.}$$

Moreover, for a given node $z\in K_{\ell }$,

(18) $$E_{\ell ,z}=⋃\left\{E\in E_{\ell }:z\in E\right\}$$

denotes the star of edges originating at z.

### 2.2 Newest vertex bisection

**Fig. 1:**
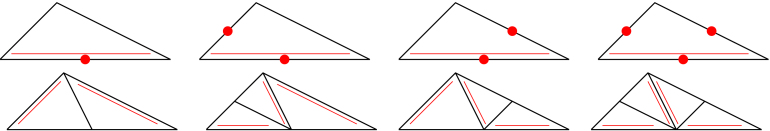
For each triangle $T\in T_{\ell }$, there is one fixed *reference edge*, indicated by the double line (left, top). Refinement of T is done by bisecting the reference edge, where its midpoint becomes a new node. The reference edges of the son triangles $T^{\prime }\in T_{\ell +1}$ are opposite to this newest vertex (left, bottom). To avoid hanging nodes, one proceeds as follows: we assume that certain edges of T, but at least the reference edge, are marked for refinement (top). Using iterated newest vertex bisection, the element is then split into 2, 3, or 4 son triangles (bottom).

Throughout, we assume that newest vertex bisection is used for mesh-refinement, see Fig. 1. Let $T_{\ell }$ be a given mesh and $M_{\ell }\subseteq E_{\ell }$ an arbitrary set of marked edges. Then,

(19) $$T_{\ell +1}=\mathrm{refine}\left(T_{\ell },M_{\ell }\right)$$

denotes the coarsest regular triangulation such that all marked edges $E\in M_{\ell }$ have been bisected. Moreover, we write

(20) $$T_{*}=\mathrm{refine}\left(T_{\ell }\right)$$

if $T_{*}$ is a finite refinement of $T_{\ell }$, i.e., there are finitely many triangulations $T_{\ell +1},\ldots ,T_{n}$ and sets of marked edges $M_{\ell }\subseteq E_{\ell },\ldots ,M_{n-1}\subseteq E_{n-1}$ such that $T_{*}=T_{n}$ and $T_{j+1}=\mathrm{refine}\left(T_{j},M_{j}\right)$ for all j=ℓ,…,n−1.

We stress that, for a fixed initial mesh $T_{0}$, only finitely many shapes of triangles $T\in T_{\ell }$ appear. In particular, only finitely many shapes of patches  (16)–(17) appear. This observation will be used below. Moreover, newest vertex bisection guarantees that any sequence $T_{\ell }$ of generated meshes with $T_{\ell +1}=\mathrm{refine}\left(T_{\ell }\right)$ is uniformly shape regular in the sense of

(21) $$\underset{\ell \in N}{\sup}\sigma \left(T_{\ell }\right)<\infty \text{,}\;\text{where }\sigma \left(T_{\ell }\right)=\underset{T\in T_{\ell }}{\max}\frac{\mathrm{diam}{\left(T\right)}^{2}}{\left|T\right|}\text{.}$$

Further details are found in  [20, Chapter 4].

### 2.3 Scott–Zhang quasi-interpolation and discrete lifting operators

Our analysis below makes heavy use of the Scott–Zhang projection $P_{\ell }:H^{1}\left(\Omega \right)\to S^{1}\left(T_{\ell }\right)$ from  [21]: For all nodes $z\in K_{\ell }$, one chooses an edge $E_{z}\in E_{\ell }$ with $z\in E_{z}$. For z∈Γ, this choice is restricted to $E_{z}\subset \Gamma$. Moreover, for $z\in {\bar{\Gamma }}_{D}$, we even enforce $E_{z}\subset {\bar{\Gamma }}_{D}$. For $w\in H^{1}\left(\Omega \right)$, $P_{\ell }w$ is then defined by

$$\left(P_{\ell }w\right)\left(z\right)≔{\langle \psi_{z},w\rangle }_{E_{z}}\text{,}$$

for a node $z\in K_{\ell }$. Here, $\psi_{z}\in L^{2}\left(E_{z}\right)$ denotes the dual basis function defined by ${\langle \psi_{z},\varphi_{z^{\prime }}\rangle }_{E_{z}}=\delta_{zz^{\prime }}$, and $\varphi_{z}\in S^{1}\left(T_{\ell }\right)$ denotes the hat function associated with $z\in K_{\ell }$. By definition, we then have the following projection properties

• $P_{\ell }W_{\ell }=W_{\ell }$ for all $W_{\ell }\in S^{1}\left(T_{\ell }\right)$,
• $\left(P_{\ell }w\right){{|}_{\Gamma }=w|}_{\Gamma }$ for all $w\in H^{1}\left(\Omega \right)$ and $W_{\ell }\in S^{1}\left(T_{\ell }\right)$ with $w{{|}_{\Gamma }=W_{\ell }|}_{\Gamma }$,
• $\left(P_{\ell }w\right){{|}_{\Gamma_{D}}=w|}_{\Gamma_{D}}$ for all $w\in H^{1}\left(\Omega \right)$ and $W_{\ell }\in S^{1}\left(T_{\ell }\right)$ with $w{{|}_{\Gamma_{D}}=W_{\ell }|}_{\Gamma_{D}}$,

i.e. the projection $P_{\ell }$ preserves discrete (Dirichlet) boundary data. Moreover, $P_{\ell }$ satisfies the following stability property

(22) $${\left\|\left(1-P_{\ell }\right)w\right\|}_{H^{1}\left(\Omega \right)}\leq C_{\mathrm{sz}}\;{\left\|\nabla w\right\|}_{L^{2}\left(\Omega \right)}\;\text{for all }w\in H^{1}\left(\Omega \right)$$

and approximation property

(23) $${\left\|\left(1-P_{\ell }\right)w\right\|}_{L^{2}\left(\Omega \right)}\leq C_{\mathrm{sz}}\;{\left\|h_{\ell }\nabla w\right\|}_{L^{2}\left(\Omega \right)}\;\text{for all }w\in H^{1}\left(\Omega \right)$$

where $C_{\mathrm{sz}}>0$ depends only on $\sigma \left(T_{\ell }\right)$ and diam(Ω). Together with the projection property onto $S^{1}\left(T_{\ell }\right)$, it is an easy consequence of the stability  (22) of $P_{\ell }$ that

(24) $${\left\|\left(1-P_{\ell }\right)w\right\|}_{H^{1}\left(\Omega \right)}=\underset{W_{\ell }\in S^{1}\left(T_{\ell }\right)}{\min}{\left\|\left(1-P_{\ell }\right)\left(w-W_{\ell }\right)\right\|}_{H^{1}\left(\Omega \right)}≲\underset{W_{\ell }\in S^{1}\left(T_{\ell }\right)}{\min}{\left\|\nabla \left(w-W_{\ell }\right)\right\|}_{L^{2}\left(\Omega \right)}$$

for all $w\in H^{1}\left(\Omega \right)$. In particular, $P_{\ell }$ is quasi-optimal in the sense of the Céa lemma with respect to ${\left\|\cdot \right\|}_{H^{1}\left(\Omega \right)}$ and ${\left\|\nabla \left(\cdot \right)\right\|}_{L^{2}\left(\Omega \right)}$, i.e.

(25) $${\left\|\left(1-P_{\ell }\right)w\right\|}_{H^{1}\left(\Omega \right)}≲\underset{W_{\ell }\in S^{1}\left(T_{\ell }\right)}{\min}{\left\|w-W_{\ell }\right\|}_{H^{1}\left(\Omega \right)}\text{,}$$

$${\left\|\nabla \left(1-P_{\ell }\right)w\right\|}_{L^{2}\left(\Omega \right)}≲\underset{W_{\ell }\in S^{1}\left(T_{\ell }\right)}{\min}{\left\|\nabla \left(w-W_{\ell }\right)\right\|}_{L^{2}\left(\Omega \right)}\text{.}$$

Moreover, $P_{\ell }$ allows to define a discrete lifting operator

(26) $$L_{\ell }≔P_{\ell }L:S^{1}\left(E_{\ell }^{\Gamma }\right)\to S^{1}\left(T_{\ell }\right)\text{,}\;\text{i.e. }L_{\ell }\left(W_{\ell }{|}_{\Gamma }\right){|}_{\Gamma }=W_{\ell }{|}_{\Gamma }\text{ for all }W_{\ell }\in S^{1}\left(T_{\ell }\right)$$

whose operator norm is uniformly bounded in terms of $\sigma \left(T_{\ell }\right)$. Here, $L\in L\left(H^{1/2}\left(\Gamma \right);H^{1}\left(\Omega \right)\right)$ denotes an arbitrary lifting operator, i.e. $\left(Lw\right){|}_{\Gamma }=w$ for all $w\in H^{1/2}\left(\Gamma \right)$, see e.g.  [22].

Finally, we put emphasis on the fact that our definition of $P_{\ell }$ also provides an operator $P_{\ell }=P_{\ell }^{\Gamma }:L^{2}\left(\Gamma \right)\to S^{1}\left(E_{\ell }^{\Gamma }\right)$ which is consistent in the sense that $\left(P_{\ell }v\right){|}_{\Gamma }=P_{\ell }^{\Gamma }\left(v{|}_{\Gamma }\right)$ for all $v\in H^{1}\left(\Omega \right)$. Using the definition of $H^{1/2}\left(\Gamma \right)$ as the trace space of $H^{1}\left(\Omega \right)$ and the stability  (22), we see

‖g^−Pℓg^‖H1/2(Γ)≔inf{‖w‖H1(Ω):w∈H1(Ω),w|Γ=g^−Pℓg^}≤inf{‖w−Pℓw‖H1(Ω):w∈H1(Ω),w|Γ=g^}≲inf{‖∇w‖L2(Ω):w∈H1(Ω),w|Γ=g^}≤inf{‖w‖H1(Ω):w∈H1(Ω),w|Γ=g^}=‖g^‖H1/2(Γ)

for all g^∈H1/2(Γ), i.e.  $P_{\ell }:H^{1/2}\left(\Gamma \right)\to S^{1}\left(E_{\ell }^{\Gamma }\right)$ is a continuous projection with respect to the $H^{1/2}$-norm. In particular, $P_{\ell }$ also provides a continuous projection $P_{\ell }=P_{\ell }^{D}:H^{1/2}\left(\Gamma_{D}\right)\to S^{1}\left(E_{\ell }^{D}\right)$, since

‖g−Pℓg‖H1/2(ΓD)≤inf{‖g^−Pℓg^‖H1/2(Γ):g^∈H1/2(Γ),g^|ΓD=g}≲inf{‖g^‖H1/2(Γ):g^∈H1/2(Γ),g^|ΓD=g}=‖g‖H1/2(ΓD)

for all $g\in H^{1/2}\left(\Gamma_{D}\right)$. As before, this definition is consistent with the previous notation of $P_{\ell }$ since (PℓΓg^)|ΓD=PℓD(g^|ΓD) for all g^∈H1/2(Γ).

## 3 A posteriori error estimation and adaptive mesh-refinement

### 3.1 Data oscillations

We start with the element data oscillations

(27) $${\mathrm{osc}}_{T,\ell }^{2}≔\underset{T\in T_{\ell }}{\sum }{\mathrm{osc}}_{T,\ell }{\left(T\right)}^{2}\text{,}\;\text{where }{\mathrm{osc}}_{T,\ell }{\left(T\right)}^{2}≔\left|T\right|\;\overset{2}{\underset{L^{2}\left(T\right)}{\left\|f-f_{T}\right\|}}\text{ for all }T\in T_{\ell }$$

and where $f_{T}≔{\left|T\right|}^{-1}\int_{T}f\;dx\in R$ denotes the integral mean over an element $T\in T_{\ell }$. These arise in the efficiency estimate for residual error estimators.

Our residual error estimator will involve the edge data oscillations

(28) $${\mathrm{osc}}_{E,\ell }^{2}≔\underset{E\in E_{\ell }^{\Omega }}{\sum }{\mathrm{osc}}_{E,\ell }{\left(E\right)}^{2}\text{,}\;\text{where }{\mathrm{osc}}_{E,\ell }{\left(E\right)}^{2}≔\left|\omega_{\ell ,E}\right|\;\overset{2}{\underset{L^{2}\left(\omega_{\ell ,E}\right)}{\left\|f-f_{\omega_{\ell ,E}}\right\|}}\text{ for all }E\in E_{\ell }^{\Omega }\text{.}$$

Here, $\omega_{\ell ,E}\subset \Omega$ is the edge patch from  (17), and $f_{\omega_{\ell ,E}}\in R$ is the corresponding integral mean of f.

For the analysis, we shall additionally need the node data oscillations

(29) $${\mathrm{osc}}_{K,\ell }^{2}≔\underset{z\in K_{\ell }^{\Omega }}{\sum }{\mathrm{osc}}_{K,\ell }{\left(z\right)}^{2}\text{,}\;\text{where }{\mathrm{osc}}_{K,\ell }{\left(z\right)}^{2}≔\left|\omega_{\ell ,z}\right|\;\overset{2}{\underset{L^{2}\left(\omega_{\ell ,z}\right)}{\left\|f-f_{\omega_{\ell ,z}}\right\|}}\text{ for all }z\in K_{\ell }^{\Omega }\text{.}$$

Here, $\omega_{\ell ,z}\subset \Omega$ is the node patch from  (16), and $f_{\omega_{\ell ,z}}\in R$ is the corresponding integral mean of f.

Moreover, the efficiency needs the Neumann data oscillations

(30) $${\mathrm{osc}}_{N,\ell }^{2}≔\underset{E\in E_{\ell }^{N}}{\sum }{\mathrm{osc}}_{N,\ell }{\left(E\right)}^{2}\text{,}\;\text{where }{\mathrm{osc}}_{N,\ell }{\left(E\right)}^{2}≔\left|E\right|\;\overset{2}{\underset{L^{2}\left(E\right)}{\left\|\phi -\phi_{E}\right\|}}\text{ for all }E\in E_{\ell }^{N}$$

and where $\phi_{E}≔{\left|E\right|}^{-1}\int_{E}\phi \;dx$ denotes the integral mean over an edge $E\in E_{\ell }^{N}$.

Finally, the approximation of the Dirichlet data $g\approx g_{\ell }$ is controlled by the Dirichlet data oscillations

(31) $${\mathrm{osc}}_{D,\ell }^{2}≔\underset{E\in E_{\ell }^{D}}{\sum }{\mathrm{osc}}_{D,\ell }{\left(E\right)}^{2}\text{,}\;\text{where }{\mathrm{osc}}_{D,\ell }{\left(E\right)}^{2}≔\left|E\right|\overset{2}{\underset{L^{2}\left(E\right)}{\left\|{\left(g-g_{\ell }\right)}^{\prime }\right\|}}\text{ for all }E\in E_{\ell }^{D}\text{.}$$

Recall that, on the 1D manifold $\Gamma_{D}$, the derivative of the nodal interpoland is the elementwise best approximation of the derivative by piecewise constants, i.e., 

(32) $${\left\|{\left(g-g_{\ell }\right)}^{\prime }\right\|}_{L^{2}\left(E\right)}=\underset{c\in R}{\min}{\left\|g^{\prime }-c\right\|}_{L^{2}\left(E\right)}\;\text{for all }E\in \overset{D}{\underset{\ell }{E}}\text{.}$$

According to the elementwise Pythagoras theorem, this implies

(33) $${\left\|{\left(g-g_{\ell }\right)}^{\prime }\right\|}_{L^{2}\left(E\right)}^{2}+{\left\|{\left(g_{\ell }-{\overset{˜}{g}}_{\ell }\right)}^{\prime }\right\|}_{L^{2}\left(E\right)}^{2}={\left\|{\left(g-{\overset{˜}{g}}_{\ell }\right)}^{\prime }\right\|}_{L^{2}\left(E\right)}^{2}\;\text{for all }{\overset{˜}{g}}_{\ell }\in S^{1}\left(E_{\ell }^{D}\right)$$

and all Dirichlet edges $E\in E_{\ell }^{D}$. This observation will be crucial in the analysis below. Moreover,  (32) yields

(34) $${\left\|h_{\ell }^{1/2}{\left(g-g_{\ell }\right)}^{\prime }\right\|}_{L^{2}\left(\Gamma_{D}\right)}=\underset{W_{\ell }\in S^{1}\left(T_{\ell }\right)}{\min}{\left\|\overset{1/2}{\underset{\ell }{h}}{\left(g-W_{\ell }{|}_{\Gamma }\right)}^{\prime }\right\|}_{L^{2}\left(\Gamma_{D}\right)}\text{.}$$

The following result is found in  [12, Lemma 2.2].

Lemma 1 *Let* $g\in H^{1}\left(\Gamma_{D}\right)$ *and let* $g_{\ell }$ *denote the nodal interpoland of* $g_{\ell }$ *on* ${\bar{\Gamma }}_{D}$ *. Then,*

(35) $${\left\|g-g_{\ell }\right\|}_{H^{1/2}\left(\Gamma_{D}\right)}\leq C_{1}\;{\mathrm{osc}}_{D,\ell }\text{,}$$

*where the constant* $C_{1}>0$ *depends only on the shape regularity constant* $\sigma \left(T_{\ell }\right)$ *and* Ω *. □*

To keep the notation simple, we extend the Dirichlet and the Neumann data oscillations from  (30) to (31) by zero to all edges $E\in E_{\ell }$, e.g.  ${\mathrm{osc}}_{D,\ell }\left(E\right)=0$ for $E\in E_{\ell }\setminus E_{\ell }^{D}$. Moreover, we will write

(36) oscT,ℓ(ωℓ,z)2=∑T∈TℓT⊂ωℓ,zoscT,ℓ(T)2resp.  oscN,ℓ(Eℓ,z)2=∑E∈EℓNE⊂Eℓ,zoscN,ℓ(E)2

to abbreviate the notation.

### 3.2 Element-based residual error estimator

Our first proposition states reliability and efficiency of the error estimator $\rho_{\ell }$ from  (5) to (6).

Proposition 2 **Reliability and efficiency of $\rho_{\ell }$**

*The error estimator* $\rho_{\ell }$ *is reliable*

(37) $${\left\|u-U_{\ell }\right\|}_{H^{1}\left(\Omega \right)}\leq C_{2}\;\rho_{\ell }$$

*and efficient*

(38) $$C_{3}^{-1}\;\rho_{\ell }\leq {\left({\left\|\nabla \left(u-U_{\ell }\right)\right\|}_{L^{2}\left(\Omega \right)}^{2}+{\mathrm{osc}}_{T,\ell }^{2}+{\mathrm{osc}}_{N,\ell }^{2}+{\mathrm{osc}}_{D,\ell }^{2}\right)}^{1/2}\text{.}$$

*The constants* $C_{2},C_{3}>0$ *depend only on the shape regularity constant* $\sigma \left(T_{\ell }\right)$ *and on* Ω*.*

Sketch of proof We consider a continuous auxiliary problem

−Δw=0in  Ω,

(39) $$w=g-g_{\ell }\;\text{ on }\Gamma_{D}\text{,}$$

$$\partial_{n}w=0\;\text{on }\Gamma_{N}\text{,}$$

with unique solution $w\in H^{1}\left(\Omega \right)$. We then have norm equivalence ${\left\|w\right\|}_{H^{1}\left(\Omega \right)}≃{\left\|g-g_{\ell }\right\|}_{H^{1/2}\left(\Gamma_{D}\right)}$ as well as $u-U_{\ell }-w\in H_{D}^{1}\left(\Omega \right)$. From this, we obtain

$${\left\|u-U_{\ell }\right\|}_{H^{1}\left(\Omega \right)}^{2}≲{\left\|\nabla \left(u-U_{\ell }-w\right)\right\|}_{L^{2}\left(\Omega \right)}^{2}+{\left\|g-g_{\ell }\right\|}_{H^{1/2}\left(\Gamma_{D}\right)}^{2}\text{.}$$

Whereas the second term is controlled by Lemma 1, the first can be handled as for homogeneous Dirichlet data, i.e. use of the Galerkin orthogonality combined with approximation estimates for a Clément-type quasi-interpolation operator. Details are found e.g. in  [6]. This proves reliability  (37).

By use of bubble functions and local scaling arguments, one obtains the estimates

$$\left|T\right|\;{\left\|f\right\|}_{L^{2}\left(T\right)}^{2}≲{\left\|\nabla \left(u-U_{\ell }\right)\right\|}_{L^{2}\left(T\right)}^{2}+{\mathrm{osc}}_{T,\ell }{\left(T\right)}^{2}+{\mathrm{osc}}_{N,\ell }\left(\partial T\cap \Gamma_{N}\right)\text{,}$$

$${\left|T\right|}^{1/2}\;{\left\|\left[\partial_{n}U_{\ell }\right]\right\|}_{L^{2}\left(E\cap \Omega \right)}^{2}≲{\left\|\nabla \left(u-U_{\ell }\right)\right\|}_{L^{2}\left(\omega_{\ell ,E}\right)}^{2}+{\mathrm{osc}}_{T,\ell }{\left(\omega_{\ell ,E}\right)}^{2}\text{,}$$

$${\left|T\right|}^{1/2}\;{\left\|\phi -\partial_{n}U_{\ell }\right\|}_{L^{2}\left(E\cap \Gamma_{N}\right)}^{2}≲{\left\|\nabla \left(u-U_{\ell }\right)\right\|}_{L^{2}\left(\omega_{\ell ,E}\right)}^{2}+{\mathrm{osc}}_{T,\ell }{\left(\omega_{\ell ,E}\right)}^{2}+{\mathrm{osc}}_{N,\ell }{\left(E\cap \Gamma_{N}\right)}^{2}\text{,}$$

where $\omega_{\ell ,E}$ denotes the edge patch of $E\in E_{\ell }$. Details are found e.g. in  [20,23]. Summing these estimates over all elements, one obtains the efficiency estimate  (38). □

Proposition 3 **Discrete local reliability of $\rho_{\ell }$**

*Let* $T_{*}=\mathrm{refine}\left(T_{\ell }\right)$ *be an arbitrary refinement of* $T_{\ell }$ *with associated Galerkin solution* $U_{*}\in S^{1}\left(T_{*}\right)$ *. Let* $R_{\ell }\left(T_{*}\right)≔T_{\ell }\setminus T_{*}$ *be the set of all elements* $T\in T_{\ell }$ *which are refined to generate* $T_{*}$ *. Then, there holds*

(40) $${\left\|U_{*}-U_{\ell }\right\|}_{H^{1}\left(\Omega \right)}\leq C_{4}\;\rho_{\ell }\left(R_{\ell }\left(T_{*}\right)\right)$$

*with some constant* $C_{4}>0$ *which depends only on* $\sigma \left(T_{\ell }\right)$ *and* Ω*.*

Proof We consider a discrete auxiliary problem

$${\langle \nabla W_{*},\nabla V_{*}\rangle }_{\Omega }=0\;\text{for all }V_{*}\in S_{D}^{1}\left(T_{*}\right)$$

with unique solution $W_{*}\in S^{1}\left(T_{*}\right)$ with $W_{*}{|}_{\Gamma_{D}}=g_{*}-g_{\ell }$. To estimate the $H^{1}$-norm of $W_{*}$ in terms of the boundary data, let $L_{*}\;:H^{1/2}\left(\Gamma \right)\to S^{1}\left(T_{*}\right)$ denote the discrete lifting operator from  (26). Let g^∗,g^ℓ∈H1/2(Γ) be arbitrary extensions of $g_{*}$ and $g_{\ell }$, respectively. Then, we have V∗=W∗−L∗(g^∗−g^ℓ)∈SD1(T∗). According to the triangle inequality and a Poincaré inequality for $V_{*}\in S_{D}^{1}\left(T_{*}\right)$, we first observe

‖W∗‖L2(Ω)≤‖V∗‖L2(Ω)+‖L∗(g^∗−g^ℓ)‖L2(Ω)≲‖∇V∗‖L2(Ω)+‖L∗(g^∗−g^ℓ)‖L2(Ω)≲‖∇W∗‖L2(Ω)+‖L∗(g^∗−g^ℓ)‖H1(Ω).

Moreover, the variational formulation for $W_{*}\in S^{1}\left(T_{*}\right)$ yields

0=〈∇W∗,∇V∗〉Ω=‖∇W∗‖L2(Ω)2−〈∇W∗,∇L∗(g^∗−g^ℓ)〉Ω,

whence by the Cauchy–Schwarz inequality

‖∇W∗‖L2(Ω)≤‖∇L∗(g^∗−g^ℓ)‖L2(Ω)≲‖g^∗−g^ℓ‖H1/2(Γ).

Altogether, this proves ‖W∗‖H1(Ω)≲‖g^∗−g^ℓ‖H1/2(Γ). Since the extensions g^∗,g^ℓ were arbitrary and by definition of the $H^{1/2}\left(\Gamma_{D}\right)$-norm, this proves

(41) $${\left\|W_{*}\right\|}_{H^{1}\left(\Omega \right)}≲{\left\|g_{*}-g_{\ell }\right\|}_{H^{1/2}\left(\Gamma_{D}\right)}≲{\left\|h_{\ell }^{1/2}{\left(g_{*}-g_{\ell }\right)}^{\prime }\right\|}_{L^{2}\left(\Gamma_{D}\right)}\text{,}$$

where we have finally used that $g_{\ell }$ is also the nodal interpoland of $g_{*}$ so that Lemma 1 applies. For an element $T\in T_{\ell }\cap T_{*}$ holds $g_{*}{|}_{\partial T\cap \Gamma_{D}}=g_{\ell }{|}_{\partial T\cap \Gamma_{D}}$, and the last term thus satisfies

$${\left\|h_{\ell }^{1/2}{\left(g_{*}-g_{\ell }\right)}^{\prime }\right\|}_{L^{2}\left(\Gamma_{D}\right)}^{2}≃\underset{T\in T_{\ell }}{\sum }{\left|T\right|}^{1/2}\overset{2}{\underset{L^{2}\left(\partial T\cap \Gamma_{D}\right)}{\left\|{\left(g_{*}-g_{\ell }\right)}^{\prime }\right\|}}=\underset{T\in R_{\ell }\left(T_{*}\right)}{\sum }{\left|T\right|}^{1/2}\overset{2}{\underset{L^{2}\left(\partial T\cap \Gamma_{D}\right)}{\left\|{\left(g_{*}-g_{\ell }\right)}^{\prime }\right\|}}\text{.}$$

With the orthogonality relation  (33) applied for $g_{*}\in S^{1}\left(T_{*}{|}_{\Gamma_{D}}\right)$, we see

$${\left\|W_{*}\right\|}_{H^{1}\left(\Omega \right)}^{2}≲\underset{T\in R_{\ell }\left(T_{*}\right)}{\sum }{\left|T\right|}^{1/2}\overset{2}{\underset{L^{2}\left(\partial T\cap \Gamma_{D}\right)}{\left\|{\left(g_{*}-g_{\ell }\right)}^{\prime }\right\|}}\leq \underset{T\in R_{\ell }\left(T_{*}\right)}{\sum }{\left|T\right|}^{1/2}\overset{2}{\underset{L^{2}\left(\partial T\cap \Gamma_{D}\right)}{\left\|{\left(g-g_{\ell }\right)}^{\prime }\right\|}}\text{.}$$

Finally, we observe $U_{*}-U_{\ell }-W_{*}\in S_{D}^{1}\left(T_{*}\right)$ with

$$\langle \nabla \left(U_{*}-U_{\ell }-W_{*}\right),\nabla V_{\ell }\rangle =0\;\text{for all }V_{\ell }\in S_{D}^{1}\left(T_{\ell }\right)\text{.}$$

Arguing as in  [1, Lemma 3.6], we see

$${\left\|\nabla \left(U_{*}-U_{\ell }-W_{*}\right)\right\|}_{L^{2}\left(\Omega \right)}^{2}≲\underset{T\in R_{\ell }\left(T_{*}\right)}{\sum }\left(\left|T\right|\;\overset{2}{\underset{L^{2}\left(T\right)}{\left\|f\right\|}}+{\left|T\right|}^{1/2}\;{\left\|\left[\partial_{n}U_{\ell }\right]\right\|}_{L^{2}\left(\partial T\cap \Omega \right)}^{2}+{\left|T\right|}^{1/2}\;{\left\|\phi -\partial_{n}U_{\ell }\right\|}_{L^{2}\left(\partial T\cap \Gamma_{N}\right)}^{2}\right)\text{.}$$

Finally, we again use the triangle inequality and the Poincaré inequality to see

$${\left\|U_{*}-U_{\ell }\right\|}_{H^{1}\left(\Omega \right)}^{2}≲{\left\|W_{*}\right\|}_{H^{1}\left(\Omega \right)}^{2}+{\left\|\nabla \left(U_{*}-U_{\ell }-W_{*}\right)\right\|}_{L^{2}\left(\Omega \right)}^{2}$$

and thus obtain the discrete local reliability  (40). The constant $C_{4}>0$ depends only on $C_{1}>0$ and on local estimates for the Scott–Zhang projection which are controlled by boundedness of $\sigma \left(T_{\ell }\right)$. □

### 3.3 Edge-based residual error estimator

In the following, we show that the edge-based estimator $\varrho_{\ell }$ from  (7) to (8) is locally equivalent to the element-based error estimator $\rho_{\ell }$ from the previous section. The main advantage is that $\varrho_{\ell }$ replaces the volume residuals

(42) $${\mathrm{res}}_{\ell }\left(T\right)≔\left|T\right|\;{\left\|f\right\|}_{L^{2}\left(T\right)}$$

by the edge oscillations ${\mathrm{osc}}_{E,\ell }$. We define the edge jump contributions

(43) $$\eta_{\ell }{\left(E\right)}^{2}≔\left\{ \begin{array}{cc} \left|E\right|\;{\left\|\left[\partial_{n}U_{\ell }\right]\right\|}_{L^{2}\left(E\right)}^{2} & \text{for }E\in E_{\ell }^{\Omega }\text{,}\\ \left|E\right|\;{\left\|\phi -\partial_{n}U_{\ell }\right\|}_{L^{2}\left(E\right)}^{2} & \text{for }E\in E_{\ell }^{N} \end{array}\right.$$

where [⋅] denotes the jump across an interior edge. Together with the edge oscillations from  (28) and the Dirichlet oscillations from  (31), our version of the residual error estimator from  (7) to (8) reads

(44) $$\varrho_{\ell }^{2}=\underset{E\in E_{\ell }}{\sum }\varrho_{\ell }{\left(E\right)}^{2}=\underset{E\in \overset{\Omega }{\underset{\ell }{E}}\cup \overset{N}{\underset{\ell }{E}}}{\sum }\eta_{\ell }{\left(E\right)}^{2}+\underset{E\in \overset{\Omega }{\underset{\ell }{E}}}{\sum }{\mathrm{osc}}_{E,\ell }{\left(E\right)}^{2}+\underset{E\in \overset{D}{\underset{\ell }{E}}}{\sum }{\mathrm{osc}}_{D,\ell }{\left(E\right)}^{2}\text{.}$$

Note that ${\mathrm{osc}}_{E,\ell }\left(E_{\ell ,z}\right)$, $\eta_{\ell }\left(E_{\ell ,z}\right)$, and ${\mathrm{res}}_{\ell }\left(\omega_{\ell ,E}\right)$ are defined analogously to  (36). The following lemma implies local equivalence of the estimators $\rho_{\ell }$ and $\varrho_{\ell }$.

Lemma 4 *The following local estimates hold:*

(i) ${\mathrm{osc}}_{T,\ell }\left(\omega_{\ell ,E}\right)\leq {\mathrm{osc}}_{E,\ell }\left(E\right)\leq C_{5}{\mathrm{res}}_{\ell }\left(\omega_{\ell ,E}\right)$ *for all* $E\in E_{\ell }^{\Omega }$*.*
(ii) ${\mathrm{res}}_{\ell }\left(\omega_{\ell ,z}\right)\leq C_{6}\left(\eta_{\ell }\left(E_{\ell ,z}\right)+{\mathrm{osc}}_{K,\ell }\left(z\right)\right)$ *for all* $z\in K_{\ell }^{\Omega }$*.*
(iii) $C_{7}^{-1}\;{\mathrm{osc}}_{E,\ell }\left(E_{\ell ,z}\right)\leq {\mathrm{osc}}_{K,\ell }\left(z\right)\leq C_{8}\;{\mathrm{osc}}_{E,\ell }\left(E_{\ell ,z}\right)$ *for all* $z\in K_{\ell }^{\Omega }$*.*

*The constants* $C_{5},C_{6},C_{7}>0$ *depend only on the shape regularity constant* $\sigma \left(T_{\ell }\right)$*, whereas* $C_{8}>0$ *depends on the use of newest vertex bisection and the initial mesh* $T_{0}$*.*

Sketch of proof The proof of (i) follows from the fact that taking the integral mean $f_{\omega }$ is the $L^{2}$ best approximation by a constant, i.e. 

$${\left\|f-f_{\omega }\right\|}_{L^{2}\left(\omega \right)}=\underset{c\in R}{\min}{\left\|f-c\right\|}_{L^{2}\left(\omega \right)}\;\text{for all measurable }\omega \subseteq \Omega \text{,}$$

and that the area of neighboring elements can only change up to $\sigma \left(T_{\ell }\right)$. The estimate (ii) is well-known and found, e.g., in[3, Section 2.2.4]. Note that (ii) essentially needs the condition that each element $T\in T_{\ell }$ has an interior node, cf. Section  2.1. The lower estimate in (iii) follows from the same arguments as (i), namely

$${\left\|f-f_{\omega_{\ell ,E}}\right\|}_{L^{2}\left(\omega_{\ell ,E}\right)}\leq {\left\|f-f_{\omega_{\ell ,z}}\right\|}_{L^{2}\left(\omega_{\ell ,E}\right)}\leq {\left\|f-f_{\omega_{\ell ,z}}\right\|}_{L^{2}\left(\omega_{\ell ,z}\right)}$$

and the fact that–up to shape regularity–only finitely many edges belong to $E_{\ell ,z}$. For f being a piecewise polynomial, the upper estimate in (iii) follows from a scaling argument since both terms, ${\mathrm{osc}}_{E,\ell }\left(E_{\ell ,z}\right)≃{\mathrm{osc}}_{K,\ell }\left(z\right)$ define seminorms on $P^{p}\left(\left\{T\in T_{\ell }:z\in T\right\}\right)$ with kernel being the constant functions. Note that the equivalence constants depend on the shape of the node patch $\omega_{\ell ,z}$, but newest vertex bisection leads only to finitely many shapes of the patches. For arbitrary $f\in L^{2}\left(\Omega \right)$, we first observe that the $T_{\ell }$-piecewise integral mean $f_{\ell }\in P^{0}\left(T_{\ell }\right)$, defined by $f_{\ell }{|}_{T}=f_{T}$ for all $T\in T_{\ell }$, satisfies ${\left(f_{\ell }\right)}_{\omega_{\ell ,E}}=f_{\omega_{\ell ,E}}$ as well as ${\left(f_{\ell }\right)}_{\omega_{\ell ,z}}=f_{\omega_{\ell ,z}}$, e.g. 

$${\left(f_{\ell }\right)}_{\omega_{\ell ,z}}=\frac{1}{\left|\omega_{\ell ,z}\right|}\int_{\omega_{\ell ,z}}f_{\ell }\;dx=\frac{1}{\left|\omega_{\ell ,z}\right|}\underset{T\subset \omega_{\ell ,z}}{\sum }\int_{T}f_{\ell }\;dx=\frac{1}{\left|\omega_{\ell ,z}\right|}\underset{T\subset \omega_{\ell ,z}}{\sum }\int_{T}f\;dx=f_{\omega_{\ell ,z}}\text{.}$$

This and the Pythagoras theorem for the integral mean $f_{\ell }$ prove

$${\left\|f-f_{\omega_{\ell ,z}}\right\|}_{L^{2}\left(\omega_{\ell ,z}\right)}^{2}={\left\|f-f_{\ell }\right\|}_{L^{2}\left(\omega_{\ell ,z}\right)}^{2}+{\left\|f_{\ell }-f_{\omega_{\ell ,z}}\right\|}_{L^{2}\left(\omega_{\ell ,z}\right)}^{2}≲\underset{E\in E_{\ell ,z}}{\sum }\overset{2}{\underset{L^{2}\left(\omega_{\ell ,E}\right)}{\left\|f-f_{\ell }\right\|}}+\underset{E\in E_{\ell ,z}}{\sum }\overset{2}{\underset{L^{2}\left(\omega_{\ell ,E}\right)}{\left\|f_{\ell }-f_{\omega_{\ell ,z}}\right\|}}=\underset{E\in E_{\ell ,z}}{\sum }\overset{2}{\underset{L^{2}\left(\omega_{\ell ,E}\right)}{\left\|f-f_{\omega_{\ell ,z}}\right\|}}\text{.}$$

Scaling with $\left|\omega_{\ell ,z}\right|≃\left|\omega_{\ell ,E}\right|$ concludes the proof. □

Proposition 5 **Reliability and efficiency of $\varrho_{\ell }$**

*The error estimator* $\varrho_{\ell }$ *is reliable*

(45) $${\left\|u-U_{\ell }\right\|}_{H^{1}\left(\Omega \right)}\leq C_{\mathrm{rel}}\;\varrho_{\ell }$$

*and efficient*

(46) $$C_{\mathrm{eff}}^{-1}\;\varrho_{\ell }\leq {\left({\left\|\nabla \left(u-U_{\ell }\right)\right\|}_{L^{2}\left(\Omega \right)}^{2}+{\mathrm{osc}}_{T,\ell }^{2}+{\mathrm{osc}}_{N,\ell }^{2}+{\mathrm{osc}}_{D,\ell }^{2}\right)}^{1/2}\text{.}$$

*The constants* $C_{\mathrm{rel}},C_{\mathrm{eff}}>0$ *depend only on* Ω*, the use of newest vertex bisection, and the initial mesh* $T_{0}$*.*

Proof With the help of the preceding lemma, we obtain equivalence $\varrho_{\ell }≃\rho_{\ell }$. Consequently, reliability and efficiency of $\varrho_{\ell }$ follow from the respective properties of the element-based estimator $\rho_{\ell }$, see Proposition 2. □

Proposition 6 **Discrete local reliability of $\varrho_{\ell }$**

*Let* $T_{*}=\mathrm{refine}\left(T_{\ell }\right)$ *be an arbitrary refinement of* $T_{\ell }$ *with associated Galerkin solution* $U_{*}\in S^{1}\left(T_{*}\right)$ *. Let* $R_{\ell }\left(T_{*}\right)≔T_{\ell }\setminus T_{*}$ *be the set of all elements* $T\in T_{\ell }$ *which are refined to generate* $T_{*}$ *and*

(47) $$R_{\ell }\left(E_{*}\right)≔\left\{E\in E_{\ell }:\exists T\in R_{\ell }\left(T_{*}\right)\;E\cap T\neq 0̸\right\}$$

*be the set of all edges which touch a refined element. Then,*

(48) $$\#R_{\ell }\left(E_{*}\right)\leq C_{\mathrm{ref}}\;\#R_{\ell }\left(T_{*}\right)$$

*and*

(49) $${\left\|U_{*}-U_{\ell }\right\|}_{H^{1}\left(\Omega \right)}\leq C_{\mathrm{dlr}}\;\varrho_{\ell }\left(R_{\ell }\left(E_{*}\right)\right)$$

*with constants* $C_{\mathrm{ref}},C_{\mathrm{dlr}}>0$ *which depend only on* Ω*, the use of newest vertex bisection, and the initial mesh* $T_{0}$*.*

Proof According to shape regularity, the number of elements which share a node $z\in K_{\ell }$ is uniformly bounded. Consequently, so is the number of edges which touch an element $T\in R_{\ell }\left(T_{*}\right)$ which will be refined. This proves the estimate $\#R_{\ell }\left(E_{*}\right)\leq C_{\mathrm{ref}}\;\#R_{\ell }\left(T_{*}\right)$. To prove  (49), we use the discrete local reliability of $\rho_{\ell }$ from Proposition 3. With the help of Lemma 4, each refinement indicator $\rho_{\ell }\left(T\right)$ for $T\in R_{\ell }\left(T_{*}\right)$ is dominated by finitely many indicators $\varrho_{\ell }\left(E\right)$ for $E\in R_{\ell }\left(E_{*}\right)$, where the number depends only on the shape regularity constant $\sigma \left(T_{\ell }\right)$. □

### 3.4 Adaptive algorithm based on Dörfler marking

Our version of the adaptive algorithm has been well-studied in the literature mainly for element-based estimators, cf. e.g.  [1].

Algorithm 7 Let adaptivity parameter 0<θ<1 and initial triangulation $T_{0}$ be given. For each ℓ=0,1,2,… do:

(i) Compute discrete solution $U_{\ell }\in S^{1}\left(T_{\ell }\right)$.
(ii) Compute refinement indicators $\varrho_{\ell }\left(E\right)$ for all $E\in E_{\ell }$.
(iii) Choose set $M_{\ell }\subseteq E_{\ell }$ with minimal cardinality such that
  (50) $$\theta \;\varrho_{\ell }^{2}\leq \varrho_{\ell }{\left(M_{\ell }\right)}^{2}\text{.}$$
(iv) Generate new mesh $T_{\ell +1}≔\mathrm{refine}\left(T_{\ell },M_{\ell }\right)$.
(v) Update counter ℓ↦ℓ+1 and go to (i).

## 4 Convergence of the adaptive algorithm

In this section, we prove a contraction property $\Delta_{\ell +1}\leq \kappa \;\Delta_{\ell }$ for some quasi-error quantity $\Delta_{\ell }≃\varrho_{\ell }^{2}$. To that end, we first introduce a locally equivalent error estimator.

Lemma 8 **Equivalent error estimator**

*Consider the extended error estimator*

(51) $${\overset{˜}{\varrho }}_{\ell }^{\;2}=\underset{E\in E_{\ell }^{\Omega }\cup E_{\ell }^{N}}{\sum }\eta_{\ell }{\left(E\right)}^{2}+\underset{E\in E_{\ell }}{\sum }{\overset{˜}{\mathrm{osc}}}_{E,\ell }{\left(E\right)}^{2}+\underset{E\in \overset{D}{\underset{\ell }{E}}}{\sum }{\mathrm{osc}}_{D,\ell }{\left(E\right)}^{2}\text{,}$$

*where the oscillation terms* ${\overset{˜}{\mathrm{osc}}}_{E,\ell }\left(E\right)$ *read*

(52) $${\overset{˜}{\mathrm{osc}}}_{E,\ell }{\left(E\right)}^{2}≔\left\{ \begin{array}{cc} {\mathrm{osc}}_{E,\ell }{\left(E\right)}^{2} & \text{for }E\in E_{\ell }^{\Omega }\text{,}\\ \left|T_{E}\right|\;{\left\|f\right\|}_{L^{2}\left(T_{E}\right)}^{2} & \text{for }E\in E_{\ell }^{\Gamma }\text{ and some }T_{E}\in T_{\ell }\text{ with }E\subset \partial T_{E}\text{.} \end{array}\right.$$

*Then, there holds equivalence in the following sense*

$$C_{9}^{-1}\;{\overset{˜}{\varrho }}_{\ell }^{\;2}\leq \varrho_{\ell }^{2}\leq {\overset{˜}{\varrho }}_{\ell }^{\;2}\;\text{and}\;\varrho_{\ell }\left(E\right)\leq {\overset{˜}{\varrho }}_{\ell }\left(E\right)\;\text{for all }E\in E_{\ell }\text{,}$$

*where* $C_{9}\geq 1$ *depends only on* $\sigma \left(T_{\ell }\right)$ *. Particularly, if* $M_{\ell }\subseteq E_{\ell }$ *satisfies the Dörfler marking*   (50)   *with* $\varrho_{\ell }$ *and* θ>0*, then* $M_{\ell }$ *satisfies the Dörfler marking with* ${\overset{˜}{\varrho }}_{\ell }$ *for some modified parameter* $0<\overset{˜}{\theta }≔\theta /C_{9}<1$*.*

Proof The estimates $\varrho_{\ell }\left(E\right)\leq {\overset{˜}{\varrho }}_{\ell }\left(E\right)\text{ for all }E\in E_{\ell }$ are obvious and imply $\varrho_{\ell }^{2}\leq {\overset{˜}{\varrho }}_{\ell }^{\;2}$. The estimate $C_{9}^{-1}\;{\overset{˜}{\varrho }}_{\ell }^{\;2}\leq \varrho_{\ell }^{2}$ follows from Lemma 4(ii) & (iii). Now, we obtain

$$\overset{˜}{\theta }\;{\overset{˜}{\varrho }}_{\ell }^{\;2}\leq \theta \;\varrho_{\ell }^{2}\leq \varrho_{\ell }{\left(M_{\ell }\right)}^{2}\leq {\overset{˜}{\varrho }}_{\ell }{\left(M_{\ell }\right)}^{2}\text{,}$$

i.e. the estimator ${\overset{˜}{\varrho }}_{\ell }$ satisfies the Dörfler marking  (50) with $\overset{˜}{\theta }≔\theta /C_{9}$. □

Lemma 9 **Estimator reduction**

*Assume that the set* $M_{\ell }\subseteq E_{\ell }$ *of marked edges satisfies the Dörfler marking*   (50)   *with* $\varrho_{\ell }$ *and some fixed parameter* 0<θ<1 *and that* $T_{\ell +1}=\mathrm{refine}\left(T_{\ell },M_{\ell }\right)$ *is obtained by local newest vertex bisection of* $T_{\ell }$ *. Then, there holds the estimator reduction estimate*

(53) $${\overset{˜}{\varrho }}_{\ell +1}^{\;2}\leq q\;{\overset{˜}{\varrho }}_{\ell }^{\;2}+C_{10}{\left\|\nabla \left(U_{\ell +1}-U_{\ell }\right)\right\|}_{L^{2}\left(\Omega \right)}^{2}$$

*with some contraction constant* q∈(0,1) *which depends only on* θ∈(0,1) *. The constant* $C_{10}>0$ *additionally depends only on the initial mesh* $T_{0}$*.*

Sketch of proof For the sake of completeness, we include the idea of the proof of  (53). To keep the notation simple, we define $\eta_{\ell }\left(E\right)=0$ for $E\in E_{\ell }^{D}$ and ${\mathrm{osc}}_{D,\ell }\left(E\right)=0$ for $E\in E_{\ell }^{\Omega }\cup E_{\ell }^{N}$ so that all contributions of ${\overset{˜}{\varrho }}_{\ell }$ are defined on the entire set of edges $E_{\ell }$.

First, we employ a triangle inequality and the Young inequality to see

$${\overset{˜}{\varrho }}_{\ell +1}^{\;2}\leq \left(1+\delta \right)\;\left(\underset{E\in E_{\ell +1}^{\Omega }}{\sum }\left|E\right|\overset{2}{\underset{L^{2}\left(E\right)}{\left\|\left[\partial_{n}U_{\ell }\right]\right\|}}+\underset{E\in E_{\ell +1}^{N}}{\sum }\left|E\right|\overset{2}{\underset{L^{2}\left(E\right)}{\left\|\phi -\partial_{n}U_{\ell }\right\|}}\right)+\left(1+\delta^{-1}\right)\left(\underset{E\in E_{\ell +1}^{\Omega }}{\sum }\left|E\right|\overset{2}{\underset{L^{2}\left(E\right)}{\left\|\left[\partial_{n}\left(U_{\ell +1}-U_{\ell }\right)\right]\right\|}}+\underset{E\in E_{\ell +1}^{N}}{\sum }\left|E\right|\overset{2}{\underset{L^{2}\left(E\right)}{\left\|\partial_{n}\left(U_{\ell +1}-U_{\ell }\right)\right\|}}\right)+{\overset{˜}{\mathrm{osc}}}_{E,\ell +1}^{2}+{\mathrm{osc}}_{D,\ell +1}^{2}\text{,}$$

where δ>0 is arbitrary. Second, a scaling argument proves

$$\underset{E\in E_{\ell +1}^{\Omega }}{\sum }\left|E\right|\overset{2}{\underset{L^{2}\left(E\right)}{\left\|\left[\partial_{n}\left(U_{\ell +1}-U_{\ell }\right)\right]\right\|}}+\underset{E\in E_{\ell +1}^{\Gamma }}{\sum }\left|E\right|\overset{2}{\underset{L^{2}\left(E\right)}{\left\|\partial_{n}\left(U_{\ell +1}-U_{\ell }\right)\right\|}}\leq C\;{\left\|\nabla \left(U_{\ell +1}-U_{\ell }\right)\right\|}_{L^{2}\left(\Omega \right)}^{2}\text{,}$$

and the constant C>0 depends only on $\sigma \left(T_{\ell }\right)$. Third, we argue as in  [1, Corollary 3.4] to see

$$\underset{E\in E_{\ell +1}^{\Omega }}{\sum }\left|E\right|\overset{2}{\underset{L^{2}\left(E\right)}{\left\|\left[\partial_{n}U_{\ell }\right]\right\|}}+\underset{E\in E_{\ell +1}^{N}}{\sum }\left|E\right|\overset{2}{\underset{L^{2}\left(E\right)}{\left\|\phi -\partial_{n}U_{\ell }\right\|}}\leq \eta_{\ell }^{2}-\frac{1}{2}\;\eta_{\ell }{\left(M_{\ell }\right)}^{2}\text{.}$$

Fourth, it is part of the proof of  [8, Theorem 5.4] that

$${\mathrm{osc}}_{D,\ell +1}^{2}\leq {\mathrm{osc}}_{D,\ell }^{2}-\frac{1}{2}\;{\mathrm{osc}}_{D,\ell }{\left(M_{\ell }\right)}^{2}\text{,}$$

which essentially follows from the orthogonality relation  (33). Fifth, in  [16, Lemma 6] it is proven that

(54) $${\overset{˜}{\mathrm{osc}}}_{E,\ell +1}^{2}\leq {\overset{˜}{\mathrm{osc}}}_{E,\ell }^{2}-\frac{1}{4}\;{\overset{˜}{\mathrm{osc}}}_{E,\ell }{\left(M_{\ell }\right)}^{2}\text{.}$$

Plugging everything together, we see

$${\overset{˜}{\varrho }}_{\ell +1}^{\;2}\leq \left(1+\delta \right)\left({\overset{˜}{\varrho }}_{\ell }^{\;2}-\frac{1}{4}\;{\overset{˜}{\varrho }}_{\ell }{\left(M_{\ell }\right)}^{2}\right)+C\left(1+\delta^{-1}\right)\;{\left\|\nabla \left(U_{\ell +1}-U_{\ell }\right)\right\|}_{L^{2}\left(\Omega \right)}^{2}\leq \left(1+\delta \right)\left(1-\overset{˜}{\theta }/4\right){\overset{˜}{\varrho }}_{\ell }^{\;2}+C\left(1+\delta^{-1}\right)\;{\left\|\nabla \left(U_{\ell +1}-U_{\ell }\right)\right\|}_{L^{2}\left(\Omega \right)}^{2}\text{,}$$

where we have used that Lemma 8 guarantees the Dörfler marking for ${\overset{˜}{\varrho }}_{\ell }$ in the second estimate. Finally, it only remains to choose δ>0 sufficiently small so that $q≔\left(1+\delta \right)\left(1-\overset{˜}{\theta }/4\right)<1$. □

The following lemma states some quasi-Galerkin orthogonality property which allows to overcome the lack of Galerkin orthogonality used in  [1].

Lemma 10 **Quasi-Galerkin orthogonality**

*Let* $T_{*}=\mathrm{refine}\left(T_{\ell }\right)$ *be an arbitrary refinement of* $T_{\ell }$ *with the associated Galerkin solution* $U_{*}\in S^{1}\left(T_{*}\right)$ *. Then,*

(55) $$2\;\left|{\langle \nabla \left(u-U_{*}\right),\nabla \left(U_{*}-U_{\ell }\right)\rangle }_{\Omega }\right|\leq \alpha \;{\left\|\nabla \left(u-U_{*}\right)\right\|}_{L^{2}\left(\Omega \right)}^{2}+\alpha^{-1}C_{\mathrm{orth}}\;{\left\|h_{\ell }^{1/2}{\left(g_{*}-g_{\ell }\right)}^{\prime }\right\|}_{L^{2}\left(\Gamma_{D}\right)}^{2}\text{,}$$

*for all* α>0*, and consequently*

(56) $$\left(1-\alpha \right){\left\|\nabla \left(u-U_{*}\right)\right\|}_{L^{2}\left(\Omega \right)}^{2}\leq {\left\|\nabla \left(u-U_{\ell }\right)\right\|}_{L^{2}\left(\Omega \right)}^{2}-{\left\|\nabla \left(U_{*}-U_{\ell }\right)\right\|}_{L^{2}\left(\Omega \right)}^{2}+\alpha^{-1}C_{\mathrm{orth}}\;{\left\|h_{\ell }^{1/2}{\left(g_{*}-g_{\ell }\right)}^{\prime }\right\|}_{L^{2}\left(\Gamma_{D}\right)}^{2}$$

*as well as*

(57) $${\left\|\nabla \left(u-U_{\ell }\right)\right\|}_{L^{2}\left(\Omega \right)}^{2}\leq \left(1+\alpha \right){\left\|\nabla \left(u-U_{*}\right)\right\|}_{L^{2}\left(\Omega \right)}^{2}+{\left\|\nabla \left(U_{*}-U_{\ell }\right)\right\|}_{L^{2}\left(\Omega \right)}^{2}+\alpha^{-1}C_{\mathrm{orth}}\;{\left\|h_{\ell }^{1/2}{\left(g_{*}-g_{\ell }\right)}^{\prime }\right\|}_{L^{2}\left(\Gamma_{D}\right)}^{2}\text{.}$$

*The constant* $C_{\mathrm{orth}}>0$ *depends only on the shape regularity of* $\sigma \left(T_{\ell }\right)$ *and* $\sigma \left(T_{*}\right)$ *and on* Ω*.*

Proof We recall the Galerkin orthogonality

$${\langle \nabla \left(u-U_{*}\right),\nabla V_{*}\rangle }_{\Omega }=0\;\text{for all }V_{*}\in S_{D}^{1}\left(T_{*}\right)\text{.}$$

Let $U_{*}^{\ell }\in S^{1}\left(T_{*}\right)$ be the unique Galerkin solution of  (4) with $U_{*}^{\ell }{|}_{\Gamma_{D}}=g_{\ell }$. We use the Galerkin orthogonality with $V_{*}=U_{*}^{\ell }-U_{\ell }\in S_{D}^{1}\left(T_{*}\right)$. This and the Young inequality allow to estimate the $L^{2}$-scalar product by

$$2\;\left|{\langle \nabla \left(u-U_{*}\right),\nabla \left(U_{*}-U_{\ell }\right)\rangle }_{\Omega }\right|=2\;\left|{\langle \nabla \left(u-U_{*}\right),\nabla \left(U_{*}-U_{*}^{\ell }\right)\rangle }_{\Omega }\right|\leq \alpha \;{\left\|\nabla \left(u-U_{*}\right)\right\|}_{L^{2}\left(\Omega \right)}^{2}+\alpha^{-1}\;{\left\|\nabla \left(U_{*}-U_{*}^{\ell }\right)\right\|}_{L^{2}\left(\Omega \right)}^{2}$$

for all α>0. To estimate the second contribution on the right-hand side, we proceed as in the proof of Proposition 3 and choose arbitrary extensions g^∗,g^ℓ∈H1/2(Γ) of the nodal interpolands $g_{*},g_{\ell }$ from $\Gamma_{D}$ to Γ. Then, we use the test function V∗=(U∗−U∗ℓ)−L∗(g^∗−g^ℓ)∈SD1(T∗) and the Galerkin orthogonalities for $U_{*},U_{*}^{\ell }\in S^{1}\left(T_{*}\right)$ to see

$$0={\langle \nabla \left(u-U_{*}^{\ell }\right),\nabla V_{*}\rangle }_{\Omega }-{\langle \nabla \left(u-U_{*}\right),\nabla V_{*}\rangle }_{\Omega }={\langle \nabla \left(U_{*}-U_{*}^{\ell }\right),\nabla V_{*}\rangle }_{\Omega }\text{.}$$

Arguing as above, we obtain

(58) $${\left\|\nabla \left(U_{*}-U_{*}^{\ell }\right)\right\|}_{L^{2}\left(\Omega \right)}≲{\left\|g_{*}-g_{\ell }\right\|}_{H^{1/2}\left(\Gamma_{D}\right)}≲{\left\|h_{\ell }^{1/2}{\left(g_{*}-g_{\ell }\right)}^{\prime }\right\|}_{L^{2}\left(\Gamma_{D}\right)}\text{.}$$

This concludes the proof of  (55).

To verify  (56)–(57), we use the identity

$${\left\|\nabla \left(u-U_{\ell }\right)\right\|}_{L^{2}\left(\Omega \right)}^{2}={\left\|\nabla \left(\left(u-U_{*}\right)+\left(U_{*}-U_{\ell }\right)\right)\right\|}_{L^{2}\left(\Omega \right)}^{2}={\left\|\nabla \left(u-U_{*}\right)\right\|}_{L^{2}\left(\Omega \right)}^{2}+2\;{\langle \nabla \left(u-U_{*}\right),\nabla \left(U_{*}-U_{\ell }\right)\rangle }_{\Omega }+{\left\|\nabla \left(U_{*}-U_{\ell }\right)\right\|}_{L^{2}\left(\Omega \right)}^{2}\text{.}$$

Rearranging the terms accordingly and use of the quasi-Galerkin orthogonality  (55) to estimate the scalar product, concludes the proof. □

Theorem 11 **Contraction of quasi-error**

*For the adaptive algorithm stated in*   Algorithm  7   *above, there are constants* γ,λ>0 *and* 0<κ<1 *such that the combined error quantity*

(59) $$\Delta_{\ell }≔{\left\|\nabla \left(u-U_{\ell }\right)\right\|}_{L^{2}\left(\Omega \right)}^{2}+\lambda \;{\mathrm{osc}}_{D,\ell }^{2}+\gamma \;{\overset{˜}{\varrho }}_{\ell }^{\;2}\geq 0$$

*satisfies a contraction property*

(60) $$\Delta_{\ell +1}\leq \kappa \;\Delta_{\ell }\;\text{for all }\ell \in N_{0}\text{.}$$

*In particular, this implies* $\lim_{\ell \to \infty }\varrho_{\ell }=0=\lim_{\ell \to \infty }{\left\|u-U_{\ell }\right\|}_{H^{1}\left(\Omega \right)}$*.*

Proof Using the quasi-Galerkin orthogonality  (56) with $T_{*}=T_{\ell +1}$, we see

$$\left(1-\alpha \right)\;{\left\|\nabla \left(u-U_{\ell +1}\right)\right\|}_{L^{2}\left(\Omega \right)}^{2}\leq {\left\|\nabla \left(u-U_{\ell }\right)\right\|}_{L^{2}\left(\Omega \right)}^{2}-{\left\|\nabla \left(U_{\ell +1}-U_{\ell }\right)\right\|}_{L^{2}\left(\Omega \right)}^{2}+\alpha^{-1}C_{\mathrm{orth}}\;{\left\|h_{\ell }^{1/2}{\left(g_{\ell +1}-g_{\ell }\right)}^{\prime }\right\|}_{L^{2}\left(\Gamma_{D}\right)}^{2}\text{.}$$

The orthogonality relation  (33) applied for $g_{\ell +1}\in S^{1}\left(T_{\ell +1}{|}_{\Gamma_{D}}\right)$ yields

$${\mathrm{osc}}_{D,\ell +1}^{2}+{\left\|h_{\ell }^{1/2}{\left(g_{\ell +1}-g_{\ell }\right)}^{\prime }\right\|}_{L^{2}\left(\Gamma_{D}\right)}^{2}\leq {\left\|h_{\ell }^{1/2}{\left(g-g_{\ell }\right)}^{\prime }\right\|}_{L^{2}\left(\Gamma_{D}\right)}^{2}={\mathrm{osc}}_{D,\ell }^{2}\text{.}$$

Together with the aforegoing estimate, we obtain

$$\left(1-\alpha \right)\;{\left\|\nabla \left(u-U_{\ell +1}\right)\right\|}_{L^{2}\left(\Omega \right)}^{2}+\alpha^{-1}C_{\mathrm{orth}}\;{\mathrm{osc}}_{D,\ell +1}^{2}\leq {\left\|\nabla \left(u-U_{\ell }\right)\right\|}_{L^{2}\left(\Omega \right)}^{2}+\alpha^{-1}C_{\mathrm{orth}}\;{\mathrm{osc}}_{D,\ell }^{2}-{\left\|\nabla \left(U_{\ell +1}-U_{\ell }\right)\right\|}_{L^{2}\left(\Omega \right)}^{2}\text{.}$$

We add the error estimator and use the estimator reduction  (53) to see, for β>0,

$$\left(1-\alpha \right)\;{\left\|\nabla \left(u-U_{\ell +1}\right)\right\|}_{L^{2}\left(\Omega \right)}^{2}+\alpha^{-1}C_{\mathrm{orth}}\;{\mathrm{osc}}_{D,\ell +1}^{2}+\beta \;{\overset{˜}{\varrho }}_{\ell +1}^{\;2}\leq {\left\|\nabla \left(u-U_{\ell }\right)\right\|}_{L^{2}\left(\Omega \right)}^{2}+\alpha^{-1}C_{\mathrm{orth}}\;{\mathrm{osc}}_{D,\ell }^{2}+\beta \;q\;{\overset{˜}{\varrho }}_{\ell }^{\;2}+\left(\beta C_{10}-1\right)\;{\left\|\nabla \left(U_{\ell +1}-U_{\ell }\right)\right\|}_{L^{2}\left(\Omega \right)}^{2}\text{.}$$

We choose β>0 sufficiently small to guarantee $\beta C_{10}-1\leq 0$. Then, we use the reliability  (45) of $\varrho_{\ell }\leq {\overset{˜}{\varrho }}_{\ell }$ in the form

$$C_{\mathrm{rel}}^{-1}\;{\left\|\nabla \left(u-U_{\ell }\right)\right\|}_{L^{2}\left(\Omega \right)}\leq C_{\mathrm{rel}}^{-1}\;{\left\|u-U_{\ell }\right\|}_{H^{1}\left(\Omega \right)}\leq {\overset{˜}{\varrho }}_{\ell }$$

to see, for ε>0,

$$\left(1-\alpha \right)\;{\left\|\nabla \left(u-U_{\ell +1}\right)\right\|}_{L^{2}\left(\Omega \right)}^{2}+\alpha^{-1}C_{\mathrm{orth}}\;{\mathrm{osc}}_{D,\ell +1}^{2}+\beta \;{\overset{˜}{\varrho }}_{\ell +1}^{\;2}\leq \left(1-\varepsilon \beta C_{\mathrm{rel}}^{-2}\right)\;{\left\|\nabla \left(u-U_{\ell }\right)\right\|}_{L^{2}\left(\Omega \right)}^{2}+\alpha^{-1}C_{\mathrm{orth}}\;{\mathrm{osc}}_{D,\ell }^{2}+\beta \left(q+\varepsilon \right)\;{\overset{˜}{\varrho }}_{\ell }^{\;2}\text{.}$$

Moreover, since ${\mathrm{osc}}_{D,\ell }$ is a contribution of ${\overset{˜}{\varrho }}_{\ell }$, we have ${\mathrm{osc}}_{D,\ell }\leq {\overset{˜}{\varrho }}_{\ell }$, whence, for δ>0,

$$\left(1-\alpha \right)\;{\left\|\nabla \left(u-U_{\ell +1}\right)\right\|}_{L^{2}\left(\Omega \right)}^{2}+\alpha^{-1}C_{\mathrm{orth}}\;{\mathrm{osc}}_{D,\ell +1}^{2}+\beta \;{\overset{˜}{\varrho }}_{\ell +1}^{\;2}\leq \left(1-\varepsilon \beta C_{\mathrm{rel}}^{-2}\right)\;{\left\|\nabla \left(u-U_{\ell }\right)\right\|}_{L^{2}\left(\Omega \right)}^{2}+\left(1-\delta \beta \right)\;\alpha^{-1}C_{\mathrm{orth}}\;{\mathrm{osc}}_{D,\ell }^{2}+\beta \left(q+\varepsilon +\delta \;\alpha^{-1}C_{\mathrm{orth}}\right)\;{\overset{˜}{\varrho }}_{\ell }^{\;2}\text{.}$$

For 0<α<1, we may now rearrange this estimate to end up with

$${\left\|\nabla \left(u-U_{\ell +1}\right)\right\|}_{L^{2}\left(\Omega \right)}^{2}+\frac{C_{\mathrm{orth}}}{\alpha \left(1-\alpha \right)}\;{\mathrm{osc}}_{D,\ell +1}^{2}+\frac{\beta }{1-\alpha }\;{\overset{˜}{\varrho }}_{\ell +1}^{\;2}\leq \frac{1-\varepsilon \beta C_{\mathrm{rel}}^{-2}}{1-\alpha }\;{\left\|\nabla \left(u-U_{\ell }\right)\right\|}_{L^{2}\left(\Omega \right)}^{2}+\left(1-\delta \beta \right)\;\frac{C_{\mathrm{orth}}}{\alpha \left(1-\alpha \right)}\;{\mathrm{osc}}_{D,\ell }^{2}+\left(q+\varepsilon +\delta \;\alpha^{-1}C_{\mathrm{orth}}\right)\;\frac{\beta }{1-\alpha }\;{\overset{˜}{\varrho }}_{\ell }^{\;2}\text{.}$$

It remains to choose the free constants 0<α,δ,ε<1, whereas β>0 has already been fixed:

• First, choose $0<\varepsilon <C_{\mathrm{rel}}^{2}/\beta$ sufficiently small to guarantee 0<q+ε<1.
• Second, choose 0<α<1 sufficiently small such that $0<\left(1-\varepsilon \beta C_{\mathrm{rel}}^{-2}\right)/\left(1-\alpha \right)<1$.
• Third, choose δ>0 sufficiently small with $0<q+\varepsilon +\delta \;\alpha^{-1}C_{\mathrm{orth}}<1$.

With γ≔β/(1−α), $\lambda ≔\alpha^{-1}\;C_{\mathrm{orth}}/\left(1-\alpha \right)$, and 0<κ<1 the maximal contraction constant of the three contributions, we conclude the proof of  (60). □

## 5 Quasi-optimality of the adaptive algorithm

### 5.1 Optimality of the marking strategy

With Theorem 11, we have seen that Dörfler marking  (50) yields a contraction of $\Delta_{\ell }≃\varrho_{\ell }^{2}$. In the following, we first observe that the Dörfler marking  (50) is not only sufficient but in some sense also necessary to obtain contraction of the estimator.

Proposition 12 **Optimality of Dörfler marking**

*Let* α>0 *and assume that the adaptivity parameter* 0<θ<1 *is sufficiently small, more precisely*

(61) $$q_{⋆}≔\frac{1-\theta \left(C_{\mathrm{dlr}}^{2}+1+\alpha^{-1}C_{\mathrm{orth}}\right)C_{\mathrm{eff}}^{2}}{1+\alpha }>0\text{.}$$

*Let* $0<q\leq q_{⋆}$ *and* $T_{*}=\mathrm{refine}\left(T_{\ell }\right)$ *and assume that*

(62) $$\left({\left\|\nabla \left(u-U_{*}\right)\right\|}_{L^{2}\left(\Omega \right)}^{2}+{\mathrm{osc}}_{E,*}^{2}+{\mathrm{osc}}_{D,*}^{2}+{\mathrm{osc}}_{N,*}^{2}\right)\leq q\;\left({\left\|\nabla \left(u-U_{\ell }\right)\right\|}_{L^{2}\left(\Omega \right)}^{2}+{\mathrm{osc}}_{E,\ell }^{2}+{\mathrm{osc}}_{D,\ell }^{2}+{\mathrm{osc}}_{N,\ell }^{2}\right)\text{.}$$

*Then, there holds the Dörfler marking for the set* $R_{\ell }\left(E_{*}\right)\subseteq E_{\ell }$ *defined in*   (47)*, i.e.*

(63) $$\theta \;\varrho_{\ell }^{2}\leq \varrho_{\ell }{\left(R_{\ell }\left(E_{*}\right)\right)}^{2}\text{.}$$

Proof We start with the elementary observation that $q\leq q_{⋆}$ is equivalent to

$$\theta \leq \frac{1-q\left(1+\alpha \right)}{\left(C_{\mathrm{dlr}}^{2}+1+\alpha^{-1}C_{\mathrm{orth}}\right)C_{\mathrm{eff}}^{2}}\text{.}$$

Using the discrete local reliability  (49) and the quasi-Galerkin orthogonality  (57), we see

$$C_{\mathrm{dlr}}^{2}\varrho_{\ell }{\left(R_{\ell }\left(E_{*}\right)\right)}^{2}\geq {\left\|\nabla \left(U_{*}-U_{\ell }\right)\right\|}_{L^{2}\left(\Omega \right)}^{2}\geq {\left\|\nabla \left(u-U_{\ell }\right)\right\|}_{L^{2}\left(\Omega \right)}^{2}-\left(1+\alpha \right)\;{\left\|\nabla \left(u-U_{*}\right)\right\|}_{L^{2}\left(\Omega \right)}^{2}-\alpha^{-1}C_{\mathrm{orth}}\;{\left\|h_{\ell }^{1/2}{\left(g_{*}-g_{\ell }\right)}^{\prime }\right\|}_{L^{2}\left(\Gamma_{D}\right)}^{2}=\left({\left\|\nabla \left(u-U_{\ell }\right)\right\|}_{L^{2}\left(\Omega \right)}^{2}+{\mathrm{osc}}_{E,\ell }^{2}+{\mathrm{osc}}_{D,\ell }^{2}+{\mathrm{osc}}_{N,\ell }^{2}\right)-\left(1+\alpha \right)\left({\left\|\nabla \left(u-U_{*}\right)\right\|}_{L^{2}\left(\Omega \right)}^{2}+{\mathrm{osc}}_{E,*}^{2}+{\mathrm{osc}}_{D,*}^{2}+{\mathrm{osc}}_{N,*}^{2}\right)-{\mathrm{osc}}_{E,\ell }^{2}-{\mathrm{osc}}_{D,\ell }^{2}-{\mathrm{osc}}_{N,\ell }^{2}+\left(1+\alpha \right)\left({\mathrm{osc}}_{E,*}^{2}+{\mathrm{osc}}_{D,*}^{2}+{\mathrm{osc}}_{N,*}^{2}\right)-\alpha^{-1}C_{\mathrm{orth}}\;{\left\|h_{\ell }^{1/2}{\left(g_{*}-g_{\ell }\right)}^{\prime }\right\|}_{L^{2}\left(\Gamma_{D}\right)}^{2}\geq \left(1-q\left(1+\alpha \right)\right)\left({\left\|\nabla \left(u-U_{\ell }\right)\right\|}_{L^{2}\left(\Omega \right)}^{2}+{\mathrm{osc}}_{E,\ell }^{2}+{\mathrm{osc}}_{D,\ell }^{2}+{\mathrm{osc}}_{N,\ell }^{2}\right)-{\mathrm{osc}}_{E,\ell }^{2}-{\mathrm{osc}}_{D,\ell }^{2}-{\mathrm{osc}}_{N,\ell }^{2}+\left(1+\alpha \right)\left({\mathrm{osc}}_{E,*}^{2}+{\mathrm{osc}}_{D,*}^{2}+{\mathrm{osc}}_{N,*}^{2}\right)-\alpha^{-1}C_{\mathrm{orth}}\;{\left\|h_{\ell }^{1/2}{\left(g_{*}-g_{\ell }\right)}^{\prime }\right\|}_{L^{2}\left(\Gamma_{D}\right)}^{2}\text{,}$$

where we have finally used Assumption  (62). As in the proof of Proposition 3, we have

$${\left\|h_{\ell }^{1/2}{\left(g_{*}-g_{\ell }\right)}^{\prime }\right\|}_{L^{2}\left(\Gamma_{D}\right)}^{2}\leq {\mathrm{osc}}_{D,\ell }{\left(R_{\ell }\left(E_{*}\right)\right)}^{2}\leq \varrho_{\ell }{\left(R_{\ell }\left(E_{*}\right)\right)}^{2}\text{.}$$

Moreover, the identities ${\mathrm{osc}}_{D,\ell }\left(E\right)={\mathrm{osc}}_{D,*}\left(E\right)$, ${\mathrm{osc}}_{E,\ell }\left(E\right)={\mathrm{osc}}_{E,*}\left(E\right)$ and ${\mathrm{osc}}_{N,\ell }\left(E\right)={\mathrm{osc}}_{N,*}\left(E\right)$ for $E\in E_{\ell }\setminus R_{\ell }\left(E_{*}\right)$ prove

(64) $${\mathrm{osc}}_{D,\ell }^{2}-{\mathrm{osc}}_{D,*}^{2}\leq {\mathrm{osc}}_{D,\ell }{\left(R_{\ell }\left(E_{*}\right)\right)}^{2}\text{,}$$

(65) $${\mathrm{osc}}_{E,\ell }^{2}-{\mathrm{osc}}_{E,*}^{2}\leq {\mathrm{osc}}_{E,\ell }{\left(R_{\ell }\left(E_{*}\right)\right)}^{2}\text{,}$$

(66) $${\mathrm{osc}}_{N,\ell }^{2}-{\mathrm{osc}}_{N,*}^{2}\leq {\mathrm{osc}}_{N,\ell }{\left(R_{\ell }\left(E_{*}\right)\right)}^{2}\text{.}$$

Note that  (65) led to the definition of $R_{\ell }\left(E_{*}\right)$ given above. Together with the efficiency  (46) and ${\mathrm{osc}}_{D,\ell }{\left(R_{\ell }\left(E_{*}\right)\right)}^{2}+{\mathrm{osc}}_{E,\ell }{\left(R_{\ell }\left(E_{*}\right)\right)}^{2}+{\mathrm{osc}}_{N,\ell }{\left(R_{\ell }\left(E_{*}\right)\right)}^{2}\leq \varrho_{\ell }{\left(R_{\ell }\left(E_{*}\right)\right)}^{2}$, we may now conclude

$$\left(C_{\mathrm{dlr}}^{2}+1+\alpha^{-1}C_{\mathrm{orth}}\right)\;\varrho_{\ell }{\left(R_{\ell }\left(E_{*}\right)\right)}^{2}\geq \left(1-q\left(1+\alpha \right)\right)\;C_{\mathrm{eff}}^{-2}\;\varrho_{\ell }^{2}\text{.}$$

This is equivalent to $\theta \;\varrho_{\ell }^{2}\leq \varrho_{\ell }{\left(R_{\ell }\left(E_{*}\right)\right)}^{2}$ and led to the definition of $q_{⋆}$. □

### 5.2 Optimality of newest vertex bisection

The quasi-optimality analysis for adaptive FEM involves two properties of the mesh-refinement which are, so far, only mathematically guaranteed for newest vertex bisection  [3,24–26] and local red-refinement with hanging nodes up to some fixed order  [27].

First, it has originally been proven in  [24] and later on improved in  [3,25,26] that the sequence of meshes defined inductively by $T_{\ell +1}≔\mathrm{refine}\left(T_{\ell },M_{\ell }\right)$ with arbitrary $M_{\ell }\subseteq E_{\ell }$ satisfies

(67) $$\#T_{\ell }-\#T_{0}\leq C_{\mathrm{nvb}}\;\overset{\ell -1}{\underset{j=0}{\sum }}\#M_{j}\;\text{for all }\ell \in N$$

with some constant $C_{\mathrm{nvb}}>0$ which depends only on $T_{0}$. This proves that the closure step in newest vertex bisection which avoids hanging nodes and leads to possible bisections of edges $E\in E_{\ell }\setminus M_{\ell }$ may not lead to arbitrary many refinements. For newest vertex bisection, the original analysis of  [24] as well as of the successors  [3,26] required that the reference edges of the initial mesh $T_{0}$ are chosen such that an interior edge $E=T_{+}\cap T_{-}\in E_{0}^{\Omega }$ is either the reference edge of both elements $T_{+},T_{-}\in T_{0}$ or of none. For the particular 2D situation, the recent work  [25] removes any assumption on $T_{0}$.

Second, for two meshes $T^{\prime }=\mathrm{refine}\left(T_{0}\right)$ and $T^{\prime \prime }=\mathrm{refine}\left(T_{0}\right)$ obtained by newest vertex bisection of the initial mesh $T_{0}$, there is a unique coarsest common refinement $T^{\prime }⊕T^{\prime \prime }=\mathrm{refine}\left(T_{0}\right)$ which is a refinement of both $T^{\prime }$ and $T^{\prime \prime }$. It is shown in  [1,5] that $T^{\prime }⊕T^{\prime \prime }$ is, in fact, the overlay of these meshes. Moreover, it holds that

(68) $$\#\left(T^{\prime }⊕T^{\prime \prime }\right)\leq \#T^{\prime }+\#T^{\prime \prime }-\#T_{0}\text{.}$$

### 5.3 Definition of the approximation class

To state the optimality result, we have to introduce the appropriate approximation class. Let

(69) $$T≔\left\{T:T=\mathrm{refine}\left(T_{0}\right)\right\}$$

be the set of all triangulations which can be obtained from $T_{0}$ by newest vertex bisection. Moreover, let

(70) $$T_{N}≔\left\{T\in T:\#T-\#T_{0}\leq N\right\}$$

be the set of triangulations which have at most N∈N elements more than the initial mesh $T_{0}$. For s>0, the approximation class $A_{s}$ has already been defined in  (12)–(13). The first step is to prove that, up to constants, nodal interpolation of the boundary data yields the best possible approximation of the exact solution.

Lemma 13 *The Galerkin solution* $U_{\ell }\in S^{1}\left(T_{\ell }\right)$ *of*   (4)   *satisfies*

(71) $${\left\|\nabla \left(u-U_{\ell }\right)\right\|}_{L^{2}\left(\Omega \right)}^{2}+{\mathrm{osc}}_{D,\ell }^{2}\leq C_{\mathrm{cea}}\left(\underset{W_{\ell }\in S^{1}\left(T_{\ell }\right)}{\inf}\overset{2}{\underset{L^{2}\left(\Omega \right)}{\left\|\nabla \left(u-W_{\ell }\right)\right\|}}+{\mathrm{osc}}_{D,\ell }^{2}\right)\text{,}$$

*where* $C_{\mathrm{cea}}>0$ *depends only on* Γ *and* $\sigma \left(T_{\ell }\right)$*.*

Proof Let g^,g^ℓ∈H1/2(Γ) denote arbitrary extensions of $g=u{|}_{\Gamma_{D}}$ resp.  $g_{\ell }$. Note that (LℓPℓg^)|ΓD=(Pℓu)|ΓD as well as (LℓPℓg^ℓ)|ΓD=gℓ, where $L_{\ell }$ denotes the discrete lifting operator from  (26). For $V_{\ell }\in S_{D}^{1}\left(T_{\ell }\right)$, we thus have Uℓ−(Vℓ+LℓPℓg^ℓ)∈SD1(Tℓ), whence

‖∇(u−Uℓ)‖L2(Ω)2=〈∇(u−Uℓ),∇(u−(Vℓ+LℓPℓg^ℓ))〉Ω

according to the Galerkin orthogonality. Therefore, the Cauchy–Schwarz inequality provides the Céa-type quasi-optimality

‖∇(u−Uℓ)‖L2(Ω)≤minVℓ∈SD1(Tℓ)‖∇(u−(Vℓ+LℓPℓg^ℓ))‖L2(Ω).

We now plug-in Vℓ=Pℓu−LℓPℓg^∈SD1(Tℓ) to see

‖∇(u−Uℓ)‖L2(Ω)≤‖∇(u−Pℓu+LℓPℓ(g^−g^ℓ))‖L2(Ω)≲‖∇(u−Pℓu)‖L2(Ω)+‖g^−g^ℓ‖H1/2(Γ).

Since the extensions g^,g^ℓ of $g,g_{\ell }$ were arbitrary, we obtain

$${\left\|\nabla \left(u-U_{\ell }\right)\right\|}_{L^{2}\left(\Omega \right)}≲{\left\|\nabla \left(u-P_{\ell }u\right)\right\|}_{L^{2}\left(\Omega \right)}+{\left\|g-g_{\ell }\right\|}_{H^{1/2}\left(\Gamma_{D}\right)}≲\underset{W_{\ell }\in S^{1}\left(T_{\ell }\right)}{\min}{\left\|\nabla \left(u-W_{\ell }\right)\right\|}_{L^{2}\left(\Omega \right)}+{\left\|\overset{1/2}{\underset{\ell }{h}}{\left(g-g_{\ell }\right)}^{\prime }\right\|}_{L^{2}\left(\Gamma_{D}\right)}=\underset{W_{\ell }\in S^{1}\left(T_{\ell }\right)}{\min}{\left\|\nabla \left(u-W_{\ell }\right)\right\|}_{L^{2}\left(\Omega \right)}+{\mathrm{osc}}_{D,\ell }$$

where we have used the quasi-optimality of the Scott–Zhang projection, see Section  2.3, and Lemma 1. Adding ${\mathrm{osc}}_{D,\ell }$ to this estimate, we conclude the proof. □

### 5.4 Quasi-optimality result

Finally, we may formally state the optimality result  (14) described in the introduction.

Theorem 14 *Suppose that the adaptivity parameter* 0<θ<1 *in*   Algorithm  7   *satisfies*   (61)   *so that the marking strategy is optimal in the sense of*   Proposition  12*. Let* $U_{\ell }\in S^{1}\left(T_{\ell }\right)$ *denote the sequence of discrete solutions generated by*   Algorithm  7*. If the given data and the corresponding weak solution of*   (2)   *satisfy* $\left(u,f,g,\phi \right)\in A_{s}$*, there holds*

(72) $${\left\|u-U_{\ell }\right\|}_{H^{1}\left(\Omega \right)}\leq C_{\mathrm{opt}}{\left(\#T_{\ell }-\#T_{0}\right)}^{-s}\text{,}$$

*i.e. each possible convergence rate* s>0 *is asymptotically achieved by AFEM. The constant* $C_{\mathrm{opt}}>0$ *depends only on* ${\left\|\left(u,f,g,\phi \right)\right\|}_{A_{s}}$*, the initial mesh* $T_{0}$*, and the adaptivity parameters.*

Proof Since the proof follows essentially the lines of  [1,5], we leave the details to the reader. For any ε>0, the definition of the approximation class $A_{s}$ guarantees some triangulation $T_{\varepsilon }\in T$ such that

$$\underset{W_{\varepsilon }\in S^{1}\left(T_{\varepsilon }\right)}{\inf}{\left(\overset{2}{\underset{L^{2}\left(\Omega \right)}{\left\|\nabla \left(u-W_{\varepsilon }\right)\right\|}}+{\left\|h_{\varepsilon }^{1/2}{\left(g-W_{\varepsilon }{|}_{\Gamma }\right)}^{\prime }\right\|}_{L^{2}\left(\Gamma_{D}\right)}^{2}+{\mathrm{osc}}_{T,\varepsilon }^{2}+{\mathrm{osc}}_{N,\varepsilon }^{2}\right)}^{1/2}\leq \varepsilon$$

and

$$\#T_{\varepsilon }-\#T_{0}≲\varepsilon^{-1/s}\text{,}$$

where the constant depends only on ${\left\|\left(u,f,g,\phi \right)\right\|}_{A_{s}}$. We now consider the overlay $T_{*}≔T_{\varepsilon }⊕T_{\ell }$. With the help of Lemma 13 as well as the elementary estimates ${\mathrm{osc}}_{T,*}\leq {\mathrm{osc}}_{T,\varepsilon }$ and ${\mathrm{osc}}_{N,*}\leq {\mathrm{osc}}_{N,\varepsilon }$, we observe

$$\Lambda_{*}≔{\left({\left\|\nabla \left(u-U_{*}\right)\right\|}_{L^{2}\left(\Omega \right)}^{2}+{\mathrm{osc}}_{D,*}^{2}+{\mathrm{osc}}_{T,*}^{2}+{\mathrm{osc}}_{N,*}^{2}\right)}^{1/2}≲\varepsilon \text{,}$$

since $S^{1}\left(T_{\varepsilon }\right)\subseteq S^{1}\left(T_{*}\right)$. Moreover, the overlay estimate  (68) predicts

$$\#R_{\ell }\left(T_{*}\right)\leq \#T_{*}-\#T_{\ell }\leq \#T_{\varepsilon }-\#T_{0}≲\varepsilon^{-1/s}\text{.}$$

Note that Lemma 4 together with reliability and efficiency of $\varrho_{*}$ yields

$$\Lambda_{*}≃{\left({\left\|\nabla \left(u-U_{*}\right)\right\|}_{L^{2}\left(\Omega \right)}^{2}+{\mathrm{osc}}_{E,*}^{2}+{\mathrm{osc}}_{D,*}^{2}+{\mathrm{osc}}_{N,*}^{2}\right)}^{1/2}\text{,}$$

where ${\mathrm{osc}}_{T,*}$ is replaced by ${\mathrm{osc}}_{E,*}$. Choosing $\varepsilon =\lambda {\left({\left\|\nabla \left(u-U_{\ell }\right)\right\|}_{L^{2}\left(\Omega \right)}^{2}+{\mathrm{osc}}_{D,\ell }^{2}+{\mathrm{osc}}_{E,\ell }^{2}+{\mathrm{osc}}_{N,\ell }^{2}\right)}^{1/2}$ with λ>0 sufficiently small, we enforce the reduction  (62) and derive that $R_{\ell }\left(E_{*}\right)\subseteq E_{\ell }$ satisfies the Dörfler marking criterion, cf.  Proposition 12. Minimality of $M_{\ell }$ thus gives

$$\#M_{\ell }\leq \#R_{\ell }\left(E_{*}\right)≲\#R_{\ell }\left(T_{*}\right)≲\varepsilon^{-1/s}≃{\left({\left\|\nabla \left(u-U_{\ell }\right)\right\|}_{L^{2}\left(\Omega \right)}^{2}+{\mathrm{osc}}_{E,\ell }^{2}+{\mathrm{osc}}_{D,\ell }^{2}+{\mathrm{osc}}_{N,\ell }^{2}\right)}^{-1/\left(2s\right)}\text{.}$$

We next note that

$$\varrho_{\ell }^{2}≃{\left\|\nabla \left(u-U_{\ell }\right)\right\|}_{L^{2}\left(\Omega \right)}^{2}+{\mathrm{osc}}_{E,\ell }^{2}+{\mathrm{osc}}_{D,\ell }^{2}+{\mathrm{osc}}_{N,\ell }^{2}≃\Delta_{\ell }$$

according to reliability and efficiency of $\varrho_{\ell }$ and the definition of the contraction quantity $\Delta_{\ell }$ in Theorem 11. Combining the last two lines, we see

$$\#M_{\ell }≲\Delta_{\ell }^{-1/\left(2s\right)}≃\varrho_{\ell }^{-1/s}\;\text{for all }\ell \in N_{0}\text{.}$$

By use of the closure estimate  (67) of newest vertex bisection, we obtain

$$\#T_{\ell }-\#T_{0}≲\overset{\ell -1}{\underset{j=0}{\sum }}\#M_{j}≲\overset{\ell -1}{\underset{j=0}{\sum }}\Delta_{j}^{-1/\left(2s\right)}\text{.}$$

Note that the contraction property  (60) of $\Delta_{j}$ implies $\Delta_{\ell }\leq \kappa^{\ell -j}\Delta_{j}$, whence $\Delta_{j}^{-1/\left(2s\right)}\leq \kappa^{\left(\ell -j\right)/\left(2s\right)}\Delta_{\ell }^{-1/\left(2s\right)}$. According to 0<κ<1 and the geometric series, this gives

$$\#T_{\ell }-\#T_{0}≲\Delta_{\ell }^{-1/\left(2s\right)}\overset{\ell -1}{\underset{j=0}{\sum }}\kappa^{\left(\ell -j\right)/\left(2s\right)}≲\Delta_{\ell }^{-1/\left(2s\right)}≃\varrho_{\ell }^{-1/s}\text{.}$$

Altogether, we may therefore conclude ${\left\|u-U_{\ell }\right\|}_{H^{1}\left(\Omega \right)}≲\varrho_{\ell }≲{\left(\#T_{\ell }-\#T_{0}\right)}^{-s}$. □

## 6 Some remarks on the 3D case

So far, we have only considered a 2D model problem  (1). In 3D, one additional difficulty is that the regularity assumption $g\in H^{1}\left(\Gamma_{D}\right)$ is not sufficient to guarantee continuity of g. Therefore, one must not use nodal interpolation to discretize $g\approx g_{\ell }$ and to define the Dirichlet data oscillations ${\mathrm{osc}}_{D,\ell }$.

If we do not use nodal interpolation to approximate $g\approx g_{\ell }$, the estimator reduction estimate  (53) becomes

(73) $$\varrho_{\ell +1}^{2}\leq q\;\varrho_{\ell }^{2}+C_{10}{\left\|U_{\ell +1}-U_{\ell }\right\|}_{H^{1}\left(\Omega \right)}^{2}\text{,}$$

where $C_{10}>0$ additionally depends on Ω. The reason for this is that the analysis provides an additional term ${\left\|g_{\ell +1}-g_{\ell }\right\|}_{H^{1/2}\left(\Gamma_{D}\right)}^{2}$ on the right-hand side of  (53) since we loose the orthogonality relation  (33) which is used in the form

$${\left\|h_{\ell +1}^{1/2}{\left(g-g_{\ell +1}\right)}^{\prime }\right\|}_{L^{2}\left(\Gamma_{D}\right)}^{2}\leq {\left\|h_{\ell +1}^{1/2}{\left(g-g_{\ell +1}\right)}^{\prime }\right\|}_{L^{2}\left(\Gamma_{D}\right)}^{2}+{\left\|h_{\ell +1}^{1/2}{\left(g_{\ell +1}-g_{\ell }\right)}^{\prime }\right\|}_{L^{2}\left(\Gamma_{D}\right)}^{2}={\left\|h_{\ell +1}^{1/2}{\left(g-g_{\ell }\right)}^{\prime }\right\|}_{L^{2}\left(\Gamma_{D}\right)}^{2}\text{.}$$

Instead, an inverse estimate and the Rellich compactness theorem yield

$${\left\|\nabla \left(U_{\ell +1}-U_{\ell }\right)\right\|}_{L^{2}\left(\Omega \right)}^{2}+{\left\|h_{\ell }^{1/2}{\left(g_{\ell +1}-g_{\ell }\right)}^{\prime }\right\|}_{L^{2}\left(\Gamma_{D}\right)}^{2}≲{\left\|\nabla \left(U_{\ell +1}-U_{\ell }\right)\right\|}_{L^{2}\left(\Omega \right)}^{2}+{\left\|g_{\ell +1}-g_{\ell }\right\|}_{H^{1/2}\left(\Gamma_{D}\right)}^{2}≃{\left\|U_{\ell +1}-U_{\ell }\right\|}_{H^{1}\left(\Omega \right)}^{2}$$

which proves  (73). Note that this estimate holds for *any* discretization of $g\approx g_{\ell }\in S^{1}\left(E_{\ell }^{D}\right)$ and even in 3D, where the arc length derivative ${\left(\cdot \right)}^{\prime }$ is replaced by the surface gradient $\nabla_{\Gamma }\left(\cdot \right)$; we refer to  [28] for the inverse estimate.

A possible choice for $g_{\ell }$ is $g_{\ell }=\Pi_{\ell }g$, where $\Pi_{\ell }:L^{2}\left(\Gamma_{D}\right)\to S^{1}\left(E_{\ell }^{D}\right)$ is the $L^{2}$-orthogonal projection  [6]. Alternatively, $g_{\ell }=P_{\ell }g$, with $P_{\ell }:H^{1/2}\to S^{1}\left(E_{\ell }^{D}\right)$ the Scott–Zhang projection is chosen  [7]. Note that newest vertex bisection of $T_{\ell }$ and hence of $E_{\ell }^{D}$ ensures that $\Pi_{\ell }$ is a stable projection with respect to the $H^{1}\left(\Gamma_{D}\right)$-norm  [25]. In  [13], we prove for either choice the approximation estimate

(74) $${\left\|g-g_{\ell }\right\|}_{H^{1/2}\left(\Gamma_{D}\right)}≲{\left\|h_{\ell }^{1/2}\nabla_{\Gamma }\left(g-g_{\ell }\right)\right\|}_{L^{2}\left(\Gamma_{D}\right)}≕{\mathrm{osc}}_{D,\ell }\text{.}$$

Moreover, we show that, for $g_{\ell }=\Pi_{\ell }g$, the a priori limit $g_{\infty }≔\lim_{\ell }g_{\ell }$ exists strongly in $H^{\alpha }\left(\Gamma_{D}\right)$ for 0≤α<1 and even weakly in $H^{1}\left(\Gamma_{D}\right)$ provided that the discrete spaces $S^{1}\left(E_{\ell }^{D}\right)$ are nested, i.e.  $S^{1}\left(E_{\ell }^{D}\right)\subseteq S^{1}\left(E_{\ell +1}^{D}\right)$ for all $\ell \in N_{0}$. Note, however, that this is always the case for adaptive mesh-refining algorithms. In particular, we have

(75) $$S^{1}\left(T_{\ell }\right)\subseteq S^{1}\left(T_{\ell +1}\right)\;\text{for all }\ell \in N_{0}\text{.}$$

In the following, we even aim to prove that nestedness  (75) implies the existence of the a priori limit $\lim_{\ell }U_{\ell }$ in $H^{1}\left(\Omega \right)$. To that end, we need the following lemma.

Lemma 15 **A priori convergence of Scott–Zhang projection**

*We recall the Scott–Zhang projection* $P_{\ell }$ *onto* $S^{1}\left(T_{\ell }\right)$ *and make the additional assumption that the edges* $E_{z}$ *are chosen appropriately, i.e. for* $\omega_{\ell ,z}\subset ⋃\left(T_{\ell }\cap T_{\ell +1}\right)$ *we ensure that the edge* $E_{z}$ *is chosen for both operators* $P_{\ell }$ *and* $P_{\ell +1}$ *. Then, the Scott–Zhang interpolands* $v_{\ell }≔P_{\ell }v\in S^{1}\left(T_{\ell }\right)$ *of arbitrary* $v\in H^{1}\left(\Omega \right)$ *converge to some a priori limit in* $H^{1}\left(\Omega \right)$*, i.e. there holds*

(76) $${\left\|P_{\infty }v-P_{\ell }v\right\|}_{H^{1}\left(\Omega \right)}\overset{\ell \to \infty }{⟶}0$$

*for a certain element* $P_{\infty }v\in S^{1}\left(T_{\infty }\right)≔\bar{{⋃}_{\ell \in N}S^{1}\left(T_{\ell }\right)}$*.*

Proof We follow the ideas from  [29] and define the following subsets of Ω:

$$\Omega_{\ell }^{0}≔⋃\left\{T\in T_{\ell }:\omega_{\ell }\left(T\right)\subset ⋃\left(\overset{\infty }{\underset{j=\ell }{⋂}}T_{j}\right)\right\}\text{,}$$

$$\Omega_{\ell }≔⋃\left\{T\in T_{\ell }:\text{There exists }k\geq 0\text{ s.t. }\omega_{\ell }\left(T\right)\text{ is at least uniformly refined in }T_{\ell +k}\right\}\text{,}$$

$$\Omega_{\ell }^{*}≔\Omega \setminus \left(\Omega_{\ell }\cup \Omega_{\ell }^{0}\right)\text{,}$$

where $\omega_{\ell }\left(\omega \right)≔⋃\left\{T\in T_{\ell }:T\cap \omega \neq 0̸\right\}$ for all measurable ω⊂Ω. According to  [29, Corollary 4.1], it holds that

(77) $$\underset{\ell \to \infty }{\lim}{\left\|\chi_{\Omega_{\ell }}h_{\ell }\right\|}_{L^{\infty }\left(\Omega \right)}=0\text{.}$$

Let ε>0 be arbitrary. Since the space $H^{2}\left(\Omega \right)$ is dense in $H^{1}\left(\Omega \right)$, we find $v_{\varepsilon }\in H^{2}\left(\Omega \right)$ such that ${\left\|v-v_{\varepsilon }\right\|}_{H^{1}\left(\Omega \right)}\leq \varepsilon$. Due to local approximation and stability properties of $P_{\ell }$, we obtain

$${\left\|\left(1-P_{\ell }\right)v\right\|}_{H^{1}\left(\Omega_{\ell }\right)}≲{\left\|\left(1-P_{\ell }\right)v_{\varepsilon }\right\|}_{H^{1}\left(\Omega_{\ell }\right)}+\varepsilon \leq {\left\|h_{\ell }\;D^{2}v_{\varepsilon }\right\|}_{L^{2}\left(\omega_{\ell }\left(\Omega_{\ell }\right)\right)}+\varepsilon \text{,}$$

cf.  [21]. By use of  (77), we may choose $\ell_{0}\in N$ sufficiently large to guarantee ${\left\|h_{\ell }\;D^{2}v_{\varepsilon }\right\|}_{L^{2}\left(\omega_{\ell }\left(\Omega_{\ell }\right)\right)}\leq {\left\|h_{\ell }\right\|}_{L^{\infty }\left(\omega_{\ell }\left(\Omega_{\ell }\right)\right)}{\left\|D^{2}v_{\varepsilon }\right\|}_{L^{2}\left(\Omega \right)}\leq \varepsilon$ for all $\ell \geq \ell_{0}$. Then, there holds

(78) $${\left\|\left(1-P_{\ell }\right)v\right\|}_{H^{1}\left(\Omega_{\ell }\right)}≲\varepsilon \;\text{for all }\ell \geq \ell_{0}\text{.}$$

There holds $\lim_{\ell \to \infty }\left|\Omega_{\ell }^{*}\right|=0$, cf.  [29, Proposition 4.2], and this provides the existence of $\ell_{1}\in N$ such that

(79) $${\left\|v\right\|}_{H^{1}\left(\omega_{\ell }\left(\Omega_{\ell }^{*}\right)\right)}\leq \varepsilon \;\text{for all }\ell \geq \ell_{1}$$

due to the non-concentration of Lebesgue functions. With these preparations, we finally aim at proving that $P_{\ell }v$ is a Cauchy sequence in $H^{1}\left(\Omega \right)$. Therefore, let $\ell \geq \max\left\{\ell_{0},\ell_{1}\right\}$ and k≥0 be arbitrary. First, we use that for any $T\in T_{\ell }$, $\left(P_{\ell }v\right){|}_{T}$ depends only on $v{|}_{\omega_{\ell }\left(T\right)}$. Then, by definition of $\Omega_{\ell }^{0}$ and our assumption on the definition of $P_{\ell }$ and $P_{\ell +k}$ on $T_{\ell }\cap T_{\ell +k}$, we obtain

(80) $${\left\|P_{\ell }v-P_{\ell +k}v\right\|}_{H^{1}\left(\Omega_{\ell }^{0}\right)}=0\text{.}$$

Second, due to the local stability of $P_{\ell }$ and  (79), there holds

(81) $${\left\|P_{\ell }v-P_{\ell +k}v\right\|}_{H^{1}\left(\Omega_{\ell }^{*}\right)}\leq {\left\|P_{\ell }v\right\|}_{H^{1}\left(\Omega_{\ell }^{*}\right)}+{\left\|P_{\ell +k}v\right\|}_{H^{1}\left(\Omega_{\ell }^{*}\right)}≲{\left\|v\right\|}_{H^{1}\left(\omega_{\ell }\left(\Omega_{\ell }^{*}\right)\right)}+{\left\|v\right\|}_{H^{1}\left(\omega_{\ell +k}\left(\Omega_{\ell }^{*}\right)\right)}\leq 2{\left\|v\right\|}_{H^{1}\left(\omega_{\ell }\left(\Omega_{\ell }^{*}\right)\right)}\leq 2\varepsilon \text{.}$$

Third, we proceed by exploiting  (78). We have

(82) $${\left\|P_{\ell }v-P_{\ell +k}v\right\|}_{H^{1}\left(\Omega_{\ell }\right)}\leq {\left\|P_{\ell }v-v\right\|}_{H^{1}\left(\Omega_{\ell }\right)}+{\left\|v-P_{\ell +k}v\right\|}_{H^{1}\left(\Omega_{\ell }\right)}≲\varepsilon \text{.}$$

Combining the estimates from  (80)–(82), we conclude ${\left\|P_{\ell }v-P_{\ell +k}v\right\|}_{H^{1}\left(\Omega \right)}≲\varepsilon$, i.e. $\left(P_{\ell }v\right)$ is a Cauchy sequence in $H^{1}\left(\Omega \right)$ and hence convergent. □

Now, we are able to prove a priori convergence of $U_{\ell }$ towards some a priori limit $u_{\infty }$.

Proposition 16 **A priori convergence of $U_{\ell }$**

*Suppose that the discrete spaces satisfy nestedness*   (75)   *and that* $U_{\ell }\in S^{1}\left(T_{\ell }\right)$ *solves*   (4)   *with* $g_{\ell }=\Pi_{\ell }g$ *and* $\Pi_{\ell }:L^{2}\left(\Gamma_{D}\right)\to S^{1}\left(E_{\ell }^{D}\right)$*the* $L^{2}$*-projection. Then, the a priori limit* $u_{\infty }≔\lim_{\ell \to \infty }U_{\ell }\in H^{1}\left(\Omega \right)$ *exists.*

Proof For $g_{\ell }\in H^{1/2}\left(\Gamma \right)$, we consider the continuous auxiliary problem

$$-\Delta w_{\ell }=0\;\text{in }\Omega \text{,}$$

$$w_{\ell }=g_{\ell }\;\text{on }\Gamma_{D}\text{,}$$

$$\partial_{n}w_{\ell }=0\;\text{on }\Gamma_{N}\text{.}$$

Let $w_{\ell }\in H^{1}\left(\Omega \right)$ be the unique (weak) solution and note that the trace g^ℓ≔wℓ|Γ∈H1/2(Γ) provides an extension of $g_{\ell }$ with

‖g^ℓ‖H1/2(Γ)≤‖wℓ‖H1(Ω)≲‖gℓ‖H1/2(ΓD)≤‖g^ℓ‖H1/2(Γ).

For arbitrary k,ℓ∈N, the same type of arguments prove

‖g^ℓ−g^k‖H1/2(Γ)≃‖gℓ−gk‖H1/2(ΓD).

Since $\left(g_{\ell }\right)$ is a Cauchy sequence in $H^{1/2}\left(\Gamma_{D}\right)$, cf.  [13], we obtain that (g^ℓ) is a Cauchy sequence in $H^{1/2}\left(\Gamma \right)$, whence convergent with limit g^∞∈H1/2(Γ).

Second, note that (Lℓg^ℓ)|ΓD=gℓ, where $L_{\ell }=P_{\ell }L$ denotes the discrete lifting from  (26). Therefore, U˜ℓ≔Uℓ−Lℓg^ℓ∈SD1(Tℓ) is the unique solution of the variational form

(83) 〈∇U˜ℓ,∇Vℓ〉Ω=〈∇u,∇Vℓ〉Ω−〈∇Lℓg^ℓ,∇Vℓ〉Ω  for all  Vℓ∈SD1(Tℓ).

Third, Lemma 15 implies

‖Lℓg^ℓ−P∞Lg^∞‖H1(Ω)≤‖Pℓ(Lg^ℓ−Lg^∞)‖H1(Ω)+‖PℓLg^∞−P∞Lg^∞‖H1(Ω)≲‖g^ℓ−g^∞‖H1/2(Γ)+‖PℓLg^∞−P∞Lg^∞‖H1(Ω)⟶ℓ→∞0.

Fourth, let ${\overset{˜}{U}}_{\ell ,\infty }\in S_{D}^{1}\left(T_{\ell }\right)$ be the unique solution of the discrete auxiliary problem

(84) 〈∇U˜ℓ,∞,∇Vℓ〉Ω=〈∇u,∇Vℓ〉Ω−〈∇P∞Lg^∞,∇Vℓ〉Ωfor all  Vℓ∈SD1(Tℓ).

Due to the nestedness of the ansatz spaces $S_{D}^{1}\left(T_{\ell }\right)$, we derive a priori convergence ${\overset{˜}{U}}_{\ell ,\infty }\overset{\ell \to \infty }{⟶}{\overset{˜}{u}}_{\infty }\in H^{1}\left(\Omega \right)$, where ${\overset{˜}{u}}_{\infty }$ denotes the Galerkin solution with respect to the closure of ${⋃}_{\ell =0}^{\infty }S_{D}^{1}\left(T_{\ell }\right)$ in $H_{0}^{1}\left(\Omega \right)$, see e.g.  [30, Lemma 6.1]. With the stability of  (83) and  (84), we obtain

‖∇(U˜ℓ,∞−U˜ℓ)‖L2(Ω)≲‖Lℓg^ℓ−P∞Lg^∞‖H1(Ω)⟶ℓ→∞0,

and therefore ${\overset{˜}{U}}_{\ell }\overset{\ell \to \infty }{⟶}{\overset{˜}{u}}_{\infty }$ in $H^{1}\left(\Omega \right)$. Finally, we conclude

Uℓ=U˜ℓ+Lℓg^ℓ⟶ℓ→∞u˜∞+P∞Lg^∞≕u∞∈H1(Ω),

which concludes the proof. □

Remark Note that Proposition 16 also holds if the Scott–Zhang projection is used to discretize $g\approx g_{\ell }=P_{\ell }g$. This immediately follows from Lemma 15, since $g_{\ell }=\left(P_{\ell }Lg\right){|}_{\Gamma_{D}}\to \left(P_{\infty }Lg\right){|}_{\Gamma_{D}}$ as ℓ→∞.  □

Theorem 17 *Suppose that either the* $L^{2}$*-projection* $g_{\ell }=\Pi_{\ell }g$ *or the Scott–Zhang operator* $g_{\ell }=P_{\ell }g$ *is used to discretize the Dirichlet data* $g\in H^{1}\left(\Gamma \right)$ *. Then,*   Algorithm  7   *guarantees* $\lim_{\ell }{\left\|u-U_{\ell }\right\|}_{H^{1}\left(\Omega \right)}=0$ *for both*  2*D and*  3*D.*

Proof With Proposition 16 and the estimator reduction  (73), we obtain

$$\varrho_{\ell +1}^{2}\leq q\;\varrho_{\ell }^{2}+\alpha_{\ell }\text{,}\;\text{where }0<q<1\;\text{and}\;\alpha_{\ell }\geq 0\text{ with }\alpha_{\ell }\overset{\ell \to \infty }{⟶}0\text{.}$$

From this and elementary calculus, we deduce estimator convergence $\lim_{\ell }\varrho_{\ell }=0$, cf.  [31] for the concept of estimator reduction. According to reliability of $\varrho_{\ell }$, this yields convergence of the adaptive algorithm. □

Note, however, that this convergence result is much weaker than the contraction result of Theorem 11. With the techniques of the present paper, it is unclear how to prove a contraction result if the additional orthogonality relation  (33) fails to hold.

## 7 Numerical experiment

### 7.1 Example with known solution

**Fig. 2:**
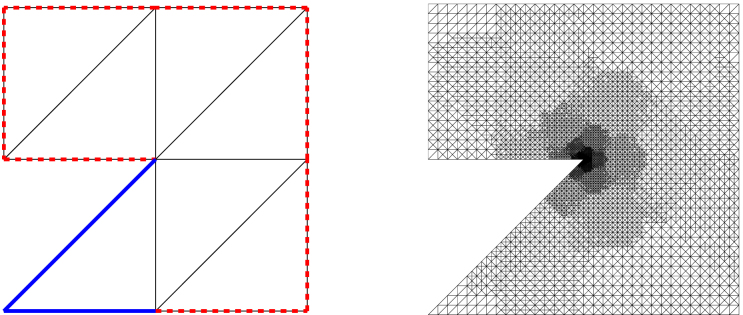
Z-shaped domain with initial mesh $T_{0}$ and adaptively generated mesh $T_{9}$ with N=10966 for θ=0.5 in Algorithm 7. The Dirichlet boundary $\Gamma_{D}$ is marked with a solid line, whereas the dashed line denotes the Neumann boundary $\Gamma \setminus \Gamma_{D}$.

On the Z-shaped domain $\Omega ={\left(-1,1\right)}^{2}\setminus \mathrm{conv}\left\{\left(0,0\right),\left(-1,-1\right),\left(0,-1\right)\right\}$, we consider the mixed boundary value problem  (1), where the partition of the boundary Γ=∂Ω into Dirichlet boundary $\Gamma_{D}$ and Neumann boundary $\Gamma_{N}$ as well as the initial mesh are shown in Fig. 2. We prescribe the exact solution u(x) in polar coordinates by

(85) $$u\left(x\right)=r^{4/7}\cos\left(4\varphi /7\right)\;\text{for }x=r\;\left(\cos\varphi ,\sin\varphi \right)\text{.}$$

Then, f=−Δu≡0, and the solution u as well as its Dirichlet data $g=u{|}_{\Gamma_{D}}$ admit a generic singularity at the reentrant corner r=0. For comparison, we implemented both marking strategies for the adaptive algorithm including the modified Dörfler criterion proposed in  [17]. We refer to the extended preprint  [19] for details.

**Fig. 3:**
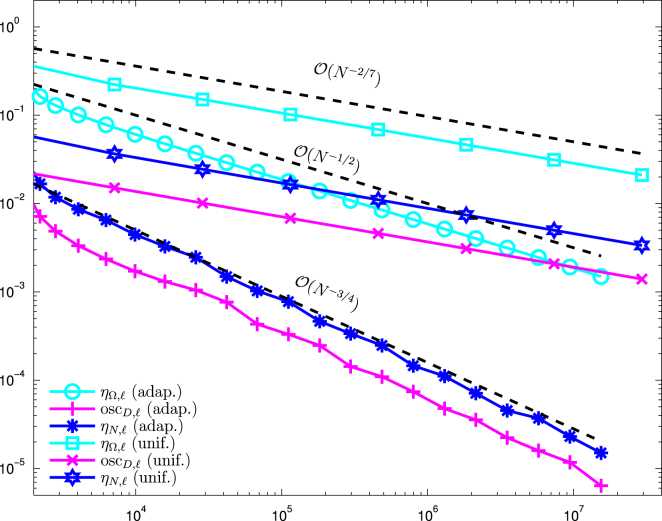
Numerical results for $\varrho_{\ell }$ for uniform and adaptive mesh-refinement with Algorithm 7 resp. the modified Dörfler marking and θ∈{0.2,0.5,0.8}, plotted over the number of elements $N=\#T_{\ell }$.

Fig. 3 shows a comparison between uniform and adaptive mesh refinement. For the algorithm based on the modified Dörfler marking, we use $\theta ≔\vartheta =\theta_{1}=\theta_{2}$. For both algorithms, we then vary the adaptivity parameter θ between 0.2 and 0.8. We observe that both adaptive algorithms lead to the optimal convergence rate $O\left(N^{-1/2}\right)$ for all choices of θ, whereas uniform refinement leads only to suboptimal convergence behavior of approximately $O\left(N^{-2/7}\right)$.

**Fig. 4:**
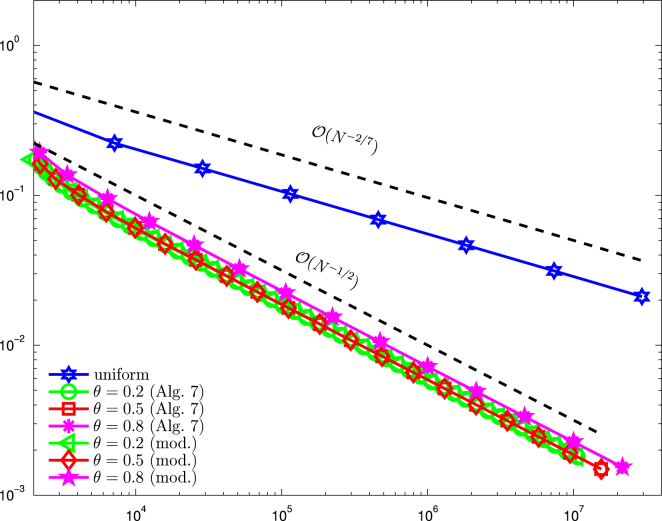
Numerical results for $\eta_{\Omega ,\ell }$, ${\mathrm{osc}}_{D,\ell }$, and $\eta_{N,\ell }$ for uniform and adaptive mesh-refinement with Algorithm 7 and θ=0.5, plotted over the number of elements $N=\#T_{\ell }$. Adaptive refinement leads to optimal convergence rates.

Note that due to f≡0, we have ${\mathrm{osc}}_{E,\ell }\equiv 0$ in this example. In Fig. 4, we compare the jump terms

$$\eta_{\Omega ,\ell }^{2}≔\underset{E\in E_{\ell }^{\Omega }}{\sum }\left|E\right|\overset{2}{\underset{L^{2}\left(E\right)}{\left\|\left[\partial_{n}U_{\ell }\right]\right\|}}\text{,}$$

the Dirichlet data oscillations ${\mathrm{osc}}_{D,\ell }$, and the Neumann jump terms

$$\eta_{N,\ell }^{2}≔\underset{E\in E_{\ell }^{N}}{\sum }\left|E\right|\overset{2}{\underset{L^{2}\left(E\right)}{\left\|\phi -\partial_{n}U_{\ell }\right\|}}$$

for uniform and adaptive refinement. Due to the corner singularity at r=0, uniform refinement leads to a suboptimal convergence behavior for $\eta_{\Omega ,\ell }$ and even for ${\mathrm{osc}}_{D,\ell }$ and $\eta_{N,\ell }$, i.e. all contributions of $\varrho_{\ell }^{2}=\eta_{\Omega ,\ell }^{2}+\eta_{N,\ell }^{2}+{\mathrm{osc}}_{D,\ell }$ show the same poor convergence rate of approximately $O\left(N^{-2/7}\right)$. For adaptive mesh-refinement, we observe that the optimal order of convergence is retained, namely $\varrho_{\ell }≃\eta_{\ell }=O\left(N^{-1/2}\right)$. Moreover, we even observe optimal convergence behavior ${\mathrm{osc}}_{D,\ell }≃\eta_{N,\ell }=O\left(N^{-3/4}\right)$ for the boundary contributions of $\varrho_{\ell }$.

Finally, in Fig. 2, the initial mesh $T_{0}$ and the adaptively generated mesh $T_{9}$ with N=10966 Elements are visualized. As expected, adaptive refinement is essentially concentrated around the reentrant corner r=0.

### 7.2 Example with unknown solution

**Fig. 5:**
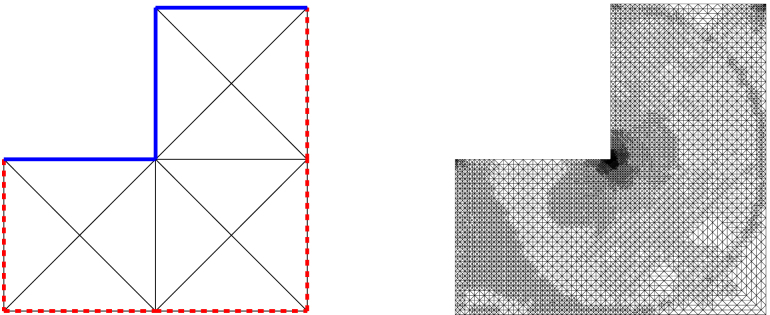
L-shaped domain with initial mesh $T_{0}$ and adaptively generated mesh $T_{9}$ with N=12177 for θ=0.5 in Algorithm 7. The Dirichlet boundary $\Gamma_{D}$ is marked with a solid line, whereas the dashed line denotes the Neumann boundary $\Gamma \setminus \Gamma_{D}$.

On the L-shaped domain $\Omega ={\left(-1,1\right)}^{2}\setminus \left(-1,0\right)\times \left(0,1\right)$, we consider the mixed boundary value problem  (1). The initial configuration with Dirichlet boundary $\Gamma_{D}$, Neumann boundary $\Gamma_{N}$, as well as the initial mesh is shown in Fig. 5. For the unknown solution $u\in H^{1}\left(\Omega \right)$, we prescribe in polar coordinates with respect to (0,0)

$$g=u{|}_{\Gamma_{D}}=r^{2/3}\sin\left(2\varphi /3\right)\;\text{on }\Gamma_{D}\text{,}$$

$$\phi =\partial_{n}u=0\;\text{on }\Gamma_{N}\text{,}$$

$$f=-\Delta u={\left|1-r\right|}^{-1/4}\;\text{in }\Omega \text{.}$$

**Fig. 6:**
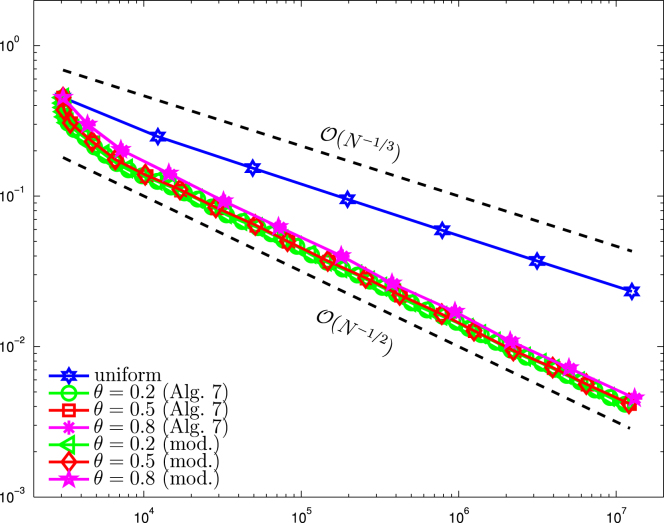
Numerical results for $\varrho_{\ell }$ for uniform and adaptive mesh-refinement with Algorithm 7 resp. the modified Dörfler marking and θ∈{0.2,0.5,0.8}, plotted over the number of elements $N=\#T_{\ell }$.

**Fig. 7:**
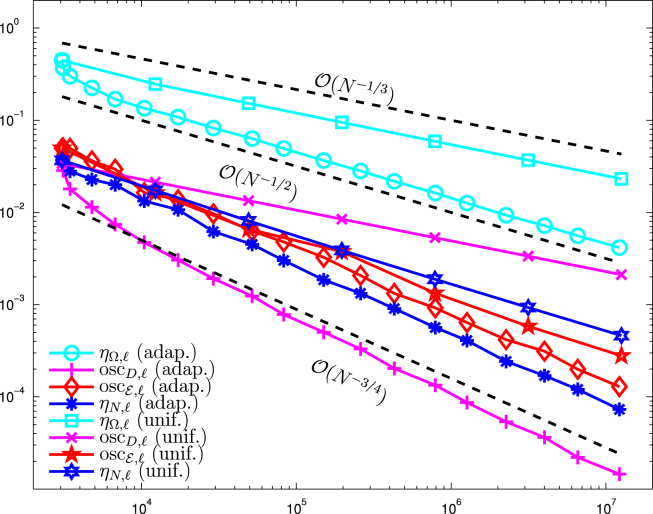
Numerical results for $\eta_{\Omega ,\ell }$, ${\mathrm{osc}}_{D,\ell }$, and $\eta_{N,\ell }$ for uniform and adaptive mesh-refinement with Algorithm 7 and θ=0.5, plotted over the number of elements $N=\#T_{\ell }$. Adaptive refinement leads to optimal convergence rates.

There holds $g\in H^{1}\left(\Gamma_{D}\right)$, $\phi \in L^{2}\left(\Gamma_{N}\right)$, and $f\in L^{2}\left(\Omega \right)$. Note that the Dirichlet data g has a singularity at the reentrant corner (0,0), whereas the volume force f is singular along the circle around (0,0) with radius r=1. Again, we compare the standard Dörfler marking strategy as well the modified Dörfler marking with the uniform approach. Fig. 6 shows a comparison between uniform and adaptive mesh refinement. The parameters $\theta =\vartheta =\theta_{1}=\theta_{2}$ are varied between 0.2 and 0.8. Both adaptive algorithms lead to optimal convergence rate $O\left(N^{-1/2}\right)$ for all choices of θ, whereas uniform refinement leads only to a suboptimal rate of $O\left(N^{-1/3}\right)$. In Fig. 7, we compare the estimator contributions which (in contrast to the previous example) include additional volume oscillations ${\mathrm{osc}}_{E,\ell }$. Due to the data singularities, as well as the singularity introduced by the change of the boundary condition, uniform refinement leads only to suboptimal convergence rates for all estimator contributions. For adaptive mesh-refinement, we observe that the optimal order of convergence is retained. This means $\varrho_{\ell }≃\eta_{\ell }=O\left(N^{-1/2}\right)$ and includes even optimal convergence behavior ${\mathrm{osc}}_{D,\ell }≃\eta_{N,\ell }=O\left(N^{-3/4}\right)$ for the boundary contributions of $\varrho_{\ell }$. In Fig. 5, one observes the adaptive refinement towards the singularity in the reentrant corner as well as the circular singularity of f and the singularities which stem from the change of boundary conditions.
